# Osteoporosis and risk of dementia among older adults: a population‑based cohort study

**DOI:** 10.1038/s41413-025-00480-7

**Published:** 2025-12-22

**Authors:** Jiangshui Wang, Shuang Wang, Cheng Jin, Xia Li, Chunbao Mo, Jing Zheng, Xiangfeng Lu, Fengchao Liang, Dongfeng Gu

**Affiliations:** 1 Key Laboratory of Cardiovascular Epidemiology, Department of Epidemiology, Fuwai Hospital, National Center for Cardiovascular Diseases, Chinese Academy of Medical Sciences and Peking Union Medical College, Beijing, China; 2 School of Public Health and Emergency Management, School of Medicine, Southern University of Science and Technology, Shenzhen, Guangdong China; 3 Shenzhen Key Laboratory of Cardiovascular Health and Precision Medicine, Southern University of Science and Technology, Shenzhen, Guangdong China; 4 Shenzhen Health Development Research and Data Management Center, Shenzhen, Guangdong China; 5 Shenzhen Institute for Innovative Applications of Healthcare Big Data, School of Public Health and Emergency Management, Southern University of Science and Technology, Shenzhen, Guangdong China; 6 SUSTech Homeostatic Medicine Institute, School of Medicine, Southern University of Science and Technology, Shenzhen, China

**Keywords:** Osteoporosis, Pathogenesis

## Abstract

Evidence on the association between osteoporosis and dementia is not fully clear. This study aimed to investigate the potential association between osteoporosis and the subsequent risk of dementia among older adults. We performed a cohort study of 176 150 community-dwelling older adults aged ≥65 years and free of cognitive impairment between 2018 and 2022 using integrated healthcare data from Shenzhen, China. Diagnoses of osteoporosis, osteoporotic fractures, and dementia were identified through linked outpatient and inpatient medical records and death registration records. Multivariate Cox proportional hazards models were used to estimate the adjusted hazard ratios (HRs) and 95% confidence intervals (CIs) of incident dementia associated with osteoporosis and osteoporotic fractures. The mean (SD) age of the total study population was 70.7 (5.4) years, and 9 605 had a previous diagnosis of osteoporosis. Over a median follow-up of 2.2 (IQR: 1.8–4.3, maximum: 5.5) years, corresponding to 505 423 person-years at risk, 1 367 incident all-cause dementia cases, including 617 Alzheimer’s disease and 298 vascular dementia cases, occurred. Physician-diagnosed osteoporosis was associated with a higher risk of all-cause dementia (HR: 1.80, 95% CI: 1.53–2.12). The increased dementia risk tended to be more prominent among patients with osteoporotic fractures (HR: 2.43, 95% CI: 1.83–3.23) than those without (HR: 1.63, 95% CI: 1.35–1.97). Results were similar for Alzheimer’s disease and vascular dementia. This study provides evidence that older adults with osteoporosis, especially those with osteoporotic fractures, have an elevated risk of incident dementia. Effective prevention and management of osteoporosis among the older population may be promising to mitigate the dual burden of osteoporosis and dementia.

## Introduction

Population aging is driving significant increases in geriatric diseases including osteoporosis and dementia in China and worldwide.^1^ Osteoporosis is a systemic, metabolic skeletal disease featuring low bone mineral density (BMD) and deteriorated bone microarchitecture, which can lead to reduced bone strength and increased risks of falls and fractures. An estimated 32.0% of adults aged ≥65 years in China had osteoporosis in 2018, and the total number of people suffering from osteoporosis is about 90 million.^2,3^ Dementia is a progressive neurodegenerative disorder that has both cognitive deficits and functional consequences and affects around 15 million (6.0%) people aged ≥60 years in China.^4^ Both these diseases can impair independence, reduce quality of life, and pose substantial burdens to patients, families, and health systems.

Although seemingly unrelated, accumulating evidence has revealed the intricate interplays between osteoporosis and dementia, which may result from not only common risk factors such as increased age, smoking, physical inactivity, shared pathological mechanisms including estrogen deficiency, inflammation, and immunology, but also intrinsic biological connections between bone and brain.^5–8^ Prior studies mainly focused on the increased risk of osteoporotic fractures among patients with dementia, while evidence on relationships between osteoporosis and subsequent dementia is still limited.^5^ Several cohort studies have implied a link between decreased BMD or diagnosed osteoporosis and incident dementia, most of which were conducted in populations of European ancestry (e.g., the Rotterdam Study, the UK Biobank).^9–13^ Considering the regional and racial differences in economic level, bone mass, and dementia incidence, evidence from China is much needed. A few cohort studies in China supported the association, but they mainly had a relatively small sample size or lacked inclusion of potential confounders such as exercise, body mass index (BMI), or comorbidities.^14,15^ In addition to prospective cohorts, studies utilizing administrative health data from Hong Kong and Taiwan regions have also revealed increased dementia risks after a diagnosis of osteoporosis or osteoporotic fracture.^16,17^ However, due to the low sensitivity of dementia diagnosis documented in electronic health records, individuals with dementia at baseline may not be excluded by prior diagnosis, thus the potential for reverse causality may exist. In addition, whether this association varies by sex remains controversial, requiring further investigation given the differences in osteoporosis and dementia prevalence between men and women.^6,17–19^ Moreover, studies examining the potential impact of osteoporotic fractures or osteoporosis medications on dementia risk in osteoporosis patients are scarce. Therefore, it is still imperative to gain more understanding in the relationship between osteoporosis and subsequent dementia. An enhanced understanding of dementia risks following osteoporosis diagnosis may help improve the management of millions of older adults with osteoporosis for dementia prevention.

In recent years, regional healthcare databases that integrate clinical and public health data have developed substantially in China,^20^ which may help to provide additional information on the relationship. In the present study, using integrated healthcare data from Shenzhen, China, we constructed a population-based cohort of community-dwelling older adults who utilized annual health management services. We aimed to investigate the association between physician-diagnosed osteoporosis and subsequent risks of dementia and its subtypes, including Alzheimer’s disease (AD) and vascular dementia (VD), among older adults without cognitive impairment at baseline. We also evaluated dementia risks for osteoporosis patients according to different clinical characteristics, including inpatient or outpatient settings, osteoporotic fracture history, and medication use.

## Results

### Baseline characteristics

This cohort study included 176 150 community-dwelling older adults with a mean age of 70.7 (SD: 5.4) years, and 55.3% were women. Of these participants, 9 605 (5.5%) had a previous diagnosis of osteoporosis. Compared to the control group, individuals with osteoporosis were older, more likely to be women, and had higher education and more comorbidities (Table 1). Among the osteoporosis group, 18.4% had a diagnosed osteoporotic fracture, and 37.7% had been prescribed osteoporotic medications (Table S1). In the propensity score matching (PSM) analyses, 9 530 older adults with osteoporosis were successfully matched with 43 913 controls, whose baseline characteristics were comparable with standardized mean differences of <0.1 (Fig. S1).

**Table 1:** Baseline characteristics of the osteoporosis and control groups

Characteristics	Full cohort analysis			Matched cohort analysis		
	Osteoporosis group (*n* = 9 605)	Control group (*n* = 166 545)	SMD	Osteoporosis group (*n* = 9 530)	Matched control group (*n* = 43 913)	SMD
Age at baseline, years	72.8 ± 6.5	70.5 ± 5.3	0.381	72.7 ± 6.5	72.3 ± 6.1	0.072
Sex						
Men	2 638 (27.5)	76 129 (45.7)	0.386	2 634 (27.6)	12 429 (28.3)	0.015
Women	6 967 (72.5)	90 416 (54.3)		6 896 (72.4)	31 484 (71.7)	
Educational level						
Junior high school or below	4 986 (51.9)	105 930 (63.6)	0.238	4 962 (52.1)	23 548 (53.6)	0.031
Senior high school or above	4 619 (48.1)	60 615 (36.4)		4 568 (47.9)	20 365 (46.4)	
Marital status			0.096			0.024
With a spouse	9 010 (93.8)	159 766 (95.9)		8 948 (93.9)	41 475 (94.4)	
Without a spouse	595 (6.2)	6 779 (4.1)		582 (6.1)	2 438 (5.6)	
Shenzhen household registration			0.616			0.066
Yes	5 919 (61.6)	53 671 (32.2)		5 846 (61.3)	25 519 (58.1)	
No	3 686 (38.4)	112 874 (67.8)		3 684 (38.7)	18 394 (41.9)	
Smoking status			0.261			0.008
Nonsmoker	8 343 (86.9)	130 378 (78.3)		8 270 (86.8)	38 042 (86.6)	
Former smoker	879 (9.2)	19 475 (11.7)		877 (9.2)	4 038 (9.2)	
Current smoker	383 (4.0)	16 692 (10.0)		383 (4.0)	1 833 (4.2)	
Exercise frequency			0.005			0.011
<1 time a week	2 111 (22.0)	36 920 (22.2)		2 085 (21.9)	9 407 (21.4)	
≥1 time a week	7 494 (78.0)	129 625 (77.8)		7 445 (78.1)	34 506 (78.6)	
BMI category, kg/m^2^			0.035			0.010
<24.0	4 996 (52.0)	87 108 (52.3)		4 947 (51.9)	22 935 (52.2)	
24.0–27.9	3 577 (37.2)	63 296 (38.0)		3 556 (37.3)	16 376 (37.3)	
≥28.0	1 032 (10.7)	16 141 (9.7)		1 027 (10.8)	4 602 (10.5)	
SBP, mmHg	132.9 ± 16.5	133.8 ± 17.4	0.057	132.9 ± 16.5	132.9 ± 16.6	<0.001
DBP, mmHg	75.5 ± 9.6	77.6 ± 10.1	0.217	75.5 ± 9.6	76.0 ± 9.6	0.047
Disease history						
Hypertension	6 318 (65.8)	80 431 (48.3)	0.359	6 243 (65.5)	27 588 (62.8)	0.056
Diabetes	3 317 (34.5)	37 999 (22.8)	0.261	3 267 (34.3)	14 018 (31.9)	0.050
Dyslipidemia	6 066 (63.2)	70 674 (42.4)	0.424	5 998 (62.9)	26 457 (60.2)	0.055
Stroke	1 807 (18.8)	13 843 (8.3)	0.310	1 746 (18.3)	6 746 (15.4)	0.079
Cancer	1 017 (10.6)	10 628 (6.4)	0.151	991 (10.4)	4 051 (9.2)	0.039
Chronic kidney disease	1 393 (14.5)	15 603 (9.4)	0.159	1 366 (14.3)	5 714 (13.0)	0.038
Depression	225 (2.3)	1 031 (0.6)	0.143	198 (2.1)	649 (1.5)	0.045

*SMD* standardized mean differences, *BMI* body mass index, *SBP* systolic blood pressure, *DBP* diastolic blood pressure

### Associations of osteoporosis with dementia risk

Over a median follow-up of 2.2 (IQR: 1.8–4.3) years and a maximum follow-up duration of 5.5 years, corresponding to 505 423 person-years at risk for dementia, 182 (1.9%) individuals with osteoporosis and 1 185 (0.7%) of controls developed all-cause dementia. The crude incidence rates of all-cause dementia were 7.64 and 2.46 per 1 000 person-years for osteoporosis and control groups, respectively (Table 2 and Fig. S2). In adjusted analyses, established risk factors of dementia partly explained the observed associations but did not eliminate the excess risk related to osteoporosis. In the minimally adjusted model taking into account only sociodemographic factors, the incidence of all-cause dementia increased by 2.18-fold (95% confidence interval [CI]: 1.85–2.56) for the osteoporosis group, compared with the control group. After further adjusting for lifestyles, BMI category, systolic blood pressure, and comorbidities in the fully adjusted model, the excessive all-cause dementia risk was still observed for the osteoporosis group [hazard ratio (HR): 1.80, 95% CI: 1.53–2.12]. Similar results were observed for AD (HR: 1.55, 95% CI: 1.20–2.00) and VD (HR: 2.13, 95% CI: 1.52–2.98). In the matched populations, the osteoporosis group still had a higher risk of all-cause dementia (HR: 1.79, 95% CI: 1.49–2.15), AD (HR: 1.59, 95% CI: 1.21–2.09), and VD (HR: 1.94, 95% CI: 1.36–2.77) compared with matched controls. Consistently higher risks of all-cause dementia in the osteoporosis group were identified in subgroup analyses stratified by demographic characteristics, socioeconomic factors, lifestyles, and comorbidities (Fig. S3). Notably, osteoporosis patients with lower education (HR: 2.24, 95% CI: 1.78–2.81) were more susceptible to the increased dementia risk compared to those with higher education (HR: 1.45, 95% CI: 1.41–1.85, *P* for interaction = 0.024).

**Table 2:** Incidence rates and hazard ratios for all-cause dementia, AD, and VD for the osteoporosis patients and control groups

Outcome	*N*	No. of events	Person-years	IR (95% CI), per 1 000 person-years	Model 1		Model 2		Model 3	
					HR (95% CI)	*P*	HR (95% CI)	*P*	HR (95% CI)	*P*
Full cohort analysis										
All-cause dementia										
Control group	166 545	1 185	481 609.0	2.46 (2.32–2.60)	ref		ref		ref	
Osteoporosis group	9 605	182	23 813.9	7.64 (6.57–8.84)	2.18 (1.85–2.56)	<0.001	2.12 (1.80–2.49)	<0.001	1.80 (1.53–2.12)	<0.001
AD										
Control group	166 545	543	482 491.3	1.13 (1.03–1.22)	ref		ref		ref	
Osteoporosis group	9 605	74	23 964.7	3.09 (2.42–3.88)	1.89 (1.47–2.43)	<0.001	1.84 (1.43–2.36)	<0.001	1.55 (1.20–2.00)	0.001
VD										
Control group	166 545	252	482 825.2	0.52 (0.46–0.59)	ref		ref		ref	
Osteoporosis group	9 605	46	23 992.5	1.92 (1.40–2.56)	2.93 (2.11–4.08)	<0.001	2.89 (2.08–4.01)	<0.001	2.13 (1.52–2.98)	<0.001
Matched cohort analysis										
All-cause dementia										
Control group	43 913	526	130 015.3	4.05 (3.71–4.41)	ref		ref		ref	
Osteoporosis group	9 530	175	23 643.4	7.40 (6.35–8.58)	1.83 (1.53–2.19)	<0.001	1.81 (1.51–2.17)	<0.001	1.79 (1.49–2.15)	<0.001
AD										
Control group	43 913	250	130 401.1	1.92 (1.69–2.17)	ref		ref		ref	
Osteoporosis group	9 530	72	23 785.3	3.03 (2.37–3.81)	1.63 (1.25–2.14)	<0.001	1.61 (1.23–2.12)	0.001	1.59 (1.21–2.09)	0.001
VD										
Control group	43 913	118	130 558.7	0.90 (0.75–1.08)	ref		ref		ref	
Osteoporosis group	9 530	45	23 812.3	1.89 (1.38–2.53)	2.01 (1.41–2.86)	<0.001	1.99 (1.39–2.83)	<0.001	1.94 (1.36–2.77)	<0.001

Model 1: adjusted for age at baseline, sex, education, marital status, and household registration; Model 2: Model 1 plus smoking, regular exercise, body mass index category, and systolic blood pressure; Model 3: Model 2 plus disease history, including hypertension, diabetes, dyslipidemia, stroke, cancer, chronic kidney disease, and depression

*AD* Alzheimer’s disease, *VD* vascular dementia, *IR* incidence rate, *CI* confidence interval, *HR* hazard ratio

### Incident dementia in osteoporosis patients by clinical characteristics

In exploratory analyses, compared with the control group, osteoporosis outpatients (HR: 1.66, 95% CI: 1.32–2.09) and osteoporosis inpatients (HR: 1.94, 95% CI: 1.56–2.40) both had an elevated risk of all-cause dementia (Table S2). Osteoporosis patients without prior osteoporotic fracture exhibited a 1.63-fold (95% CI: 1.35–1.97) higher risk of all-cause dementia, while the excess risk for those with an osteoporotic fracture reached 2.43-fold (95% CI: 1.83–3.23, Table 3). In addition, a significant increase in the all-cause dementia risk was observed among osteoporosis patients without medication use (HR: 1.94, 95% CI: 1.63–2.31), but not in those who received medications (HR: 1.25, 95% CI: 0.84–1.84, Table S3). Similar results were found for dementia subtypes and in PSM analyses.

**Table 3:** Incidence rates and hazard ratios for all-cause dementia, AD, and VD in osteoporosis patients with and without prior osteoporotic fractures

Outcome	*N*	No. of events	Person-years	IR (95% CI), per 1 000 person-years	Model 1		Model 2		Model 3	
					HR (95% CI)	*P*	HR (95% CI)	*P*	HR (95% CI)	*P*
Full cohort analysis										
All-cause dementia										
Control group	166 545	1 185	481 609.0	2.46 (2.32–2.60)	ref		ref		ref	
Without osteoporotic fractures	7 833	130	19 463.5	6.68 (5.58–7.93)	1.96 (1.63–2.37)	<0.001	1.93 (1.60–2.32)	<0.001	1.63 (1.35–1.97)	<0.001
With osteoporotic fractures	1 772	52	4 350.4	11.95 (8.93–15.67)	3.01 (2.27–3.99)	<0.001	2.86 (2.15–3.80)	<0.001	2.43 (1.83–3.23)	<0.001
AD										
Control group	166 545	543	482 491.3	1.13 (1.03–1.22)	ref		ref		ref	
Without osteoporotic fractures	7 833	53	19 574.8	2.71 (2.03–3.54)	1.71 (1.28–2.29)	<0.001	1.67 (1.25–2.23)	<0.001	1.41 (1.05–1.89)	0.022
With osteoporotic fractures	1 772	21	4 389.9	4.78 (2.96–7.31)	2.60 (1.67–4.05)	<0.001	2.47 (1.59–3.86)	<0.001	2.08 (1.33–3.25)	0.001
VD										
Control group	166 545	252	482 825.2	0.52 (0.46–0.59)	ref		ref		ref	
Without osteoporotic fractures	7 833	28	19 602.0	1.43 (0.95–2.06)	2.22 (1.48–3.31)	<0.001	2.20 (1.47–3.29)	<0.001	1.60 (1.07–2.41)	0.023
With osteoporotic fractures	1 772	18	4 390.5	4.10 (2.43–6.48)	6.11 (3.72–10.03)	<0.001	5.82 (3.54–9.57)	<0.001	4.37 (2.65–7.21)	<0.001
Matched cohort analysis										
All-cause dementia										
Control group	43 913	526	130 015.3	4.05 (3.71–4.41)	ref		ref		ref	
Without osteoporotic fractures	7 770	124	19 318.2	6.42 (5.34–7.65)	1.62 (1.32–1.99)	<0.001	1.61 (1.32–1.98)	<0.001	1.59 (1.30–1.96)	<0.001
With osteoporotic fractures	1 760	51	4 325.2	11.79 (8.78–15.50)	2.65 (1.96–3.59)	<0.001	2.57 (1.90–3.48)	<0.001	2.57 (1.90–3.46)	<0.001
AD										
Control group	43 913	250	130 401.1	1.92 (1.69–2.17)	ref		ref		ref	
Without osteoporotic fractures	7 770	52	19 420.7	2.68 (2.00–3.51)	1.48 (1.09–2.01)	0.012	1.47 (1.08–2.00)	0.014	1.45 (1.07–1.97)	0.018
With osteoporotic fractures	1 760	20	4 364.6	4.58 (2.80–7.08)	2.25 (1.41–3.61)	0.001	2.16 (1.35–3.46)	0.001	2.14 (1.34–3.41)	0.001
VD										
Control group	43 913	118	130 558.7	0.90 (0.75–1.08)	ref		ref		ref	
Without osteoporotic fractures	7 770	27	19 448.0	1.39 (0.91–2.02)	1.48 (0.96–2.28)	0.073	1.48 (0.96–2.28)	0.073	1.43 (0.93–2.21)	0.102
With osteoporotic fractures	1 760	18	4 364.3	4.12 (2.44–6.52)	4.26 (2.54–7.16)	<0.001	4.09 (2.43–6.88)	<0.001	4.14 (2.46–6.96)	<0.001

Osteoporotic fractures include pelvic, hip, wrist, spine, or proximal humeral fractures. Model 1: adjusted for age at baseline, sex, education, marital status, and household registration; Model 2: Model 1 plus smoking, regular exercise, body mass index category, and systolic blood pressure; Model 3: Model 2 plus disease history, including hypertension, diabetes, dyslipidemia, stroke, cancer, chronic kidney disease, and depression

*AD* Alzheimer’s disease, *VD* vascular dementia, *IR* incidence rate, *CI* confidence interval, *HR* hazard ratio

### Sensitivity analyses

All sensitivity analyses yielded qualitatively similar results as the primary analysis, albeit with a slightly changed magnitude of effects. In Fine-Gray models treating death unrelated to dementia as competing events, osteoporosis patients still had a higher risk of dementia (HR: 1.76, 95% CI: 1.48–2.08) compared with controls (Table S4). Using a more stringent definition of dementia, further adjusting for the number of clinical visits, and restricting to participants who had clinical visits before baseline also yielded slightly smaller relative risk estimates (Tables S5–7). Results did not change materially in other sensitivity analyses: excluding incident dementia cases that occurred within 1 year, excluding those with benzodiazepine or anticholinergic drugs use within 3 months before baseline, excluding individuals with stroke history, excluding those with thyroid disease history, and further adjusting for antihypertensive, antidiabetic, lipid-lowering, and antiplatelet medications and proton pump inhibitors use (Tables S8–10). When restricting the control group to older adults without osteoporosis based on BMD assessed by quantitative ultrasound, the magnitude of the association between osteoporosis and dementia risk was generally larger (Table S10). The *E*-value of 3.0 suggests that a relatively strong unmeasured confounder would be required to nullify the association, supporting the robustness of our findings (Fig. S4). In the time-varying Cox regression models, older adults with prevalent osteoporosis and incident osteoporosis both represented a higher risk of subsequent dementia compared with controls (Table S11).

## Discussion

In this population-based retrospective cohort of 176 150 Chinese older adults, we found that older adults with osteoporosis exhibited an increased risk of incident dementia and its subtypes (i.e., AD and VD) after controlling for various potential confounders. Additionally, these observed associations persisted when stratified by demographic, socioeconomic, lifestyle, and comorbidity subgroups and in multiple sensitivity analyses. Importantly, the increased risk tended to be more prominent among older adults with osteoporotic fractures. Osteoporosis medications may attenuate the increased risk.

The hypothesis that osteoporosis may be associated with increased dementia risks has been examined by several retrospective cohorts using administrative health databases and conventional prospective cohorts. To our knowledge, four studies have used administrative data to investigate osteoporosis and the subsequent risk of dementia. Specifically, a study using health insurance data from Taiwan, China included 23 941 patients with osteoporosis and 47 579 matched controls and reported a higher risk of dementia following an osteoporosis diagnosis (HR: 1.46, 95% CI: 1.37–1.56).^16^ A German study correlated diagnosed osteoporosis with a 1.2-fold increase in dementia risk based on 29 983 patients with osteoporosis and 1:1 matched controls in general practices.^21^ Using a national health screening database in Korea, a study of 78 994 patients with osteoporosis and matched controls found a relationship between osteoporosis and increased occurrence of AD (HR: 1.27, 95% CI: 1.22–1.32).^18^ Another recently published study based on 546 709 individuals from the Korean National Health Insurance Service database also showed that osteoporosis increased the risk of developing early-onset and late-onset dementia by 18%.^22^ Although these studies had impressive sample sizes through using administrative databases, due to the lack of information on cognitive screening and lifestyles in electronic health records, the researchers excluded prevalent dementia cases only by prior dementia diagnosis and could not consider important confounders like physical activity. As dementia is often underdiagnosed and lifestyle factors are recognized risk factors for osteoporosis and dementia, the potential for reverse causality and residual confounding may exist. Using integrated healthcare data from a megacity, we tried to address these limitations by focusing on older adults who attended geriatric health management, including cognitive screenings, which enabled us to exclude individuals with potential cognitive impairment at baseline and enhanced our ability to control confounding, and we still found significantly higher risks of dementia, AD and VD following physician-recognized osteoporosis. These findings are also in line with several prospective cohorts showing that low BMD or osteoporosis was independently associated with higher dementia risks^9–15,23^ and a recent prediction study identifying diagnosed osteoporosis as an important predictor of AD.^24^ Differences in the strength of association may result from heterogeneity in population vulnerability, diagnostic practices, exposure definitions, and dementia ascertainment. Taken together, epidemiological evidence from observational cohorts and real-world data of different ancestries and regions collectively suggests that osteoporosis patients may be a clinically meaningful risk group for the subsequent development of dementia. Previous studies have revealed discordant results on whether this association exists only among women.^18,21,23^ Our study supports that men with osteoporosis are also at increased risk of dementia. It should also be noted that the increased risk of dementia was more prominent among individuals with lower education, which may result from less health literacy and later access to diagnosis and treatment. These results should bring our attention to the necessity of monitoring cognitive function among older adults with osteoporosis, both men and women, and especially among vulnerable populations.

As osteoporosis and dementia are both common geriatric diseases, it is conceivable that shared risk factors such as increased age, smoking, physical inactivity, and comorbidities including prevalent diabetes may explain some of the observed associations.^10^ However, adjustment for these factors did not eliminate the associations between osteoporosis and dementia in the Cox regression, Fine-Gray models, and PSM analyses. In addition, recent Mendelian randomization analyses, using genetic variants as quasi-randomized instruments, have also suggested causal associations between osteoporosis and AD or structural changes in specific brain regions.^25–27^ Prior studies have suggested multiple pathogenetic mechanisms that could serve as potential explanations, including estrogen exposure, vitamin D deficiency, oxidative stress, chronic inflammation, immunological factors, mutual risk genes, gut microbiota, and circulating bone-derived modulators.^5–8^ In fact, the “bone-brain axis” has been supported by several mechanistic studies.^28^ Bone can function as an endocrine organ by secreting multiple osteokines that regulate body metabolism and brain function. Several bone-derived signaling molecules linking bone and brain have been found, such as osteocalcin, osteopontin, Lipocalin 2, fibroblast growth factor 23, and sclerostin.^5,8,29,30^ For example, sclerostin, mainly secreted from mature osteocytes in bone, is one of the major antagonists of the Wnt-β-catenin pathway, which is involved in bone metabolism and numerous nervous system processes. Animal studies elucidated that elevated secretion of sclerostin could cross the blood-brain barrier, inhibit Wnt-β-catenin signaling in the brain, and impair cognitive function in mice.^28^ Clinical studies also showed that elevated plasma sclerostin was associated with high amyloid-β load in the human brain.^31^ The underlying mechanisms mediating the complex bone-brain interorgan connections need to be further unraveled, which may have untapped potential in early detection or therapeutic target identification for dementia.

Osteoporotic fracture is an indication of severe osteoporosis. Another interesting result of our study is that we found osteoporosis patients with a history of fractures are even more susceptible to increased dementia risk, which is consistent with previous data showing the associations between fractures and subsequent risks of dementia.^17,32,33^ This is potentially attributed to the inflammatory response and reactive oxidative stress during the fracture-healing process.^33^ Several inflammatory markers increase following fractures, which are associated with an increased risk of dementia.^34^ After fractures, the reactive oxygen species also generate excessively, which will trigger endothelial dysfunction and consequently contribute to the development of VD and increase the amounts of amyloid-beta peptides.^32^ Moreover, bone loss accelerates after fractures due to immobilization, mechanical unloading, blood supply damage, and other mechanisms,^35^ which also acts as an aggregator for cognitive decline.^36^ These findings are concerning, given the large prevalence, low screening and treatment rates of osteoporosis in China and worldwide.^2^ Notably, cognitive impairment and dementia also amplify the risk of falls and fractures and induce additional bone loss, thus engendering a detrimental cycle.^5,36^ In this context, early prevention, screening, and diagnosis of osteoporosis, effective management to prevent falls and fractures, and full‑cycle, comprehensive geriatric evaluations may be needed to mitigate the dual burden of osteoporosis and dementia among older adults. Our study also hinted that the excess dementia risk associated with osteoporosis might be lessened with the use of osteoporosis medications, which has also been suggested in a previous study.^16^ While current understanding of the potential neuroprotective effects of anti-osteoporosis medications is limited, studies have proposed that bisphosphonates may exert beneficial effects through preventing fractures and inhibiting protein prenylation in the mevalonate pathway, a process involved in the pathogenesis of AD.^37^ However, due to the low treatment rate of osteoporosis, we were unable to analyze the impact of specific drug classes separately. These findings should therefore be viewed as hypothesis-generating, and further interventional studies are needed to address whether pharmacologic or non-pharmacologic treatments for osteoporosis can mitigate adverse outcomes of dementia in older persons.^5,6,8^

One of the major strengths of our study is that the use of routinely collected healthcare data ensured a large sample size and reflected contemporary real-world clinical settings. Specifically, we were able to include older adults without cognition impairment at baseline via information on geriatric health management and accounted for clinical characteristics of osteoporosis patients using linked electronic health records. Another strength is that multiple sensitivity analyses to minimize bias were conducted and led to consistent results. Still, several limitations should be acknowledged. First, the observational nature of the study precludes us from inferring causality. Although we tried to control known confounders based on previous studies, we could not adjust for sex steroids, serum vitamin D, and APOE genotypes due to the scarcity of such data. Second, data on BMD measured by dual X-ray absorptiometry were unavailable, limiting our ability to evaluate the severity of osteoporosis by BMD. Third, as the absence of a diagnosis does not assure the absence of the disease, some individuals in the control group may also have undiagnosed osteoporosis or osteoporotic fractures (e.g., vertebral fractures). However, this misclassification of exposure status is expected to lead to an underestimation of the observed association. As osteoporosis is often unrecognized until symptoms like low back pain or fractures arise, our findings may be interpreted as that osteoporosis severe enough to require medical service is associated with subsequent dementia. Fourth, diagnostic bias may occur when osteoporosis patients engage more frequently with the healthcare system than controls, increasing their chance of receiving a dementia diagnosis. While we restricted to individuals with previous clinical visits and further adjusted for the number of clinical visits, residual bias may still persist. Fifth, the relatively short follow-up duration limits our ability to assess the long-term dementia risk. Finally, the inclusion of only older adults who completed cognitive screening may have resulted in an overrepresentation of more health-conscious individuals, and as this study was conducted in a megacity in China, caution should be exercised in extrapolating these findings to rural populations or other regions.

In conclusion, our study provides evidence that older adults with osteoporosis had an increased risk of incident dementia than those without osteoporosis. Patients with an osteoporotic fracture history appeared to face an even higher excess risk. These results highlight the need to prevent and diagnose osteoporosis early, protect osteoporosis patients from fractures, and include cognitive assessment as an integral part of routine management.

## Materials and methods

### Data source

**Fig. 1:**
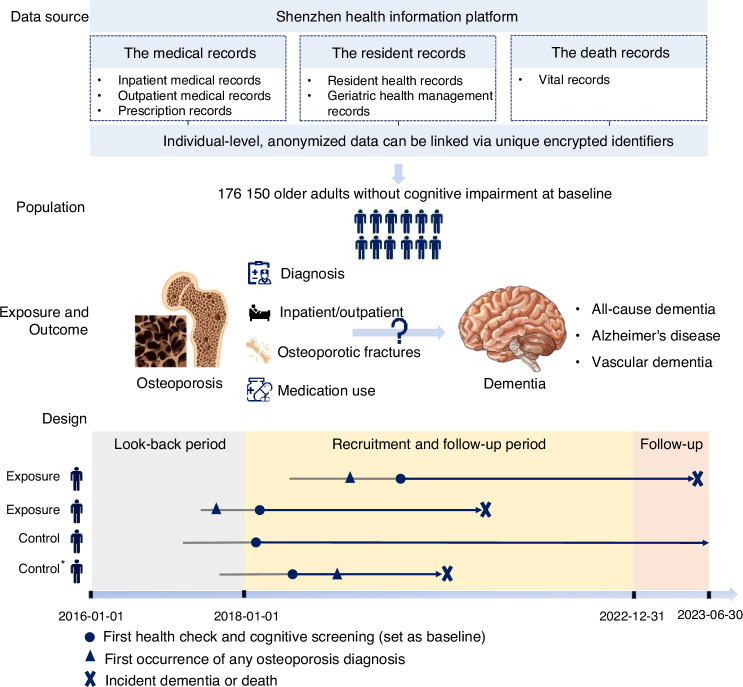
Data source and study design. Using an integrated healthcare database in Shenzhen, China, we attempted to conduct a population-based retrospective cohort study. We selected 176 150 community-dwelling older adults aged ≥65 years who attended a health check-up and screened negative for cognitive impairment between 2018 and 2022 from the geriatric health management records. A 2-year “look-back” period from 2016 to 2017 was set to extract disease history. We identified osteoporosis, osteoporotic fractures, and dementia cases through linking to outpatient and inpatient medical records, and death registration records. Information on covariates was obtained from various sources, including resident health records and electronic medical records. The first health check-up with cognitive function tested during recruitment was set as the baseline date. Participants were then split into two groups according to whether they had a prior osteoporosis diagnosis: the osteoporosis group (i.e., exposure group) and the control group. Follow-up for incident dementia began at the baseline date for both groups and continued until the earliest dementia diagnosis, death, loss to follow-up, or end of follow-up (June 30th, 2023). ^*^Incident osteoporosis during the follow-up period was treated as a time-varying exposure in a sensitivity analysis

We performed a population-based retrospective cohort study utilizing data from the Shenzhen Health Information Platform, which has been described elsewhere.^38–40^ Briefly, this regional healthcare database was designed for compiling and integrating health-related data from medical and health institutions in Shenzhen, covering community health service centers, hospitals at all levels, and public health agencies. Professional data engineers extract healthcare data from institutional databases according to specific standards, transform them into harmonized formats, and perform quality control checks for completeness, consistency, timeliness, and adherence to data standards before uploading the data to the platform.^41^ Anonymized data across different sources can be linked via unique encrypted identifiers. Specifically, for the present study, we retrieved data from January 1st, 2016 through June 30th, 2023 from various sources, including resident health records, geriatric health management records, electronic medical records of outpatient and inpatient visits, prescription records, and vital records. The data source and study design of this study are shown in Fig. 1. This study was approved by the medical ethical review committee of the Southern University of Science and Technology (Approval No. 20240215). As all data were de-identified, informed consent from participants was waived.

### Study population and exposure assessment

**Fig. 2:**
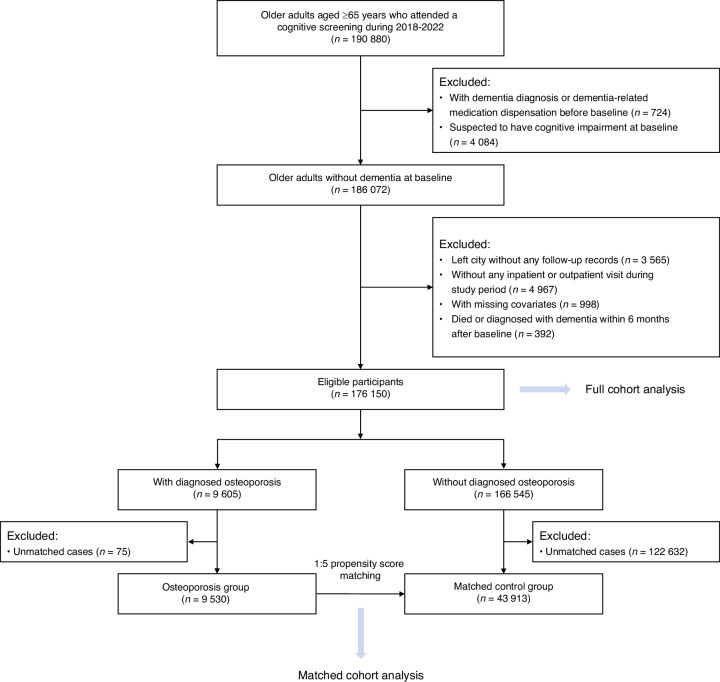
Flowchart of study population selection. In the full cohort analysis, we first selected older adults aged ≥65 years who attended a cognitive screening between 2018 and 2022 (*n* = 190 880). We excluded individuals with dementia diagnosis or dementia-related medication dispensation (donepezil, rivastigmine, galantamine, memantine) before baseline (*n* = 724), individuals with suspected cognitive impairment at baseline (*n* = 4 084), who left the city without any follow-up records (*n* = 3 565), without any inpatient or outpatient visit during study period (*n* = 4 967), with data missing on covariates (*n* = 998), died or diagnosed with dementia within 6 months following baseline date (*n* = 392). Eventually, 176 150 eligible participants were included in the final analyses. In the matched cohort analysis, 9 530 older adults with osteoporosis were successfully matched with 43 913 controls by propensity score matching

In Shenzhen, permanent residents aged ≥65 years are eligible for public annual health management services, which consist of health assessment and physical examinations at the community health service centers and are free of charge. In the present study, older adults aged ≥65 years with a geriatric health management record were included if they attended a health checkup and finished a cognitive screening by Ascertain Dementia 8-item Questionnaire (AD8) between January 1st, 2018 and December 31st, 2022 (*n* = 190 880). AD8 is a brief screening tool for cognitive impairment with good diagnostic accuracy, which has been validated in primary health care centers.^42^ Participants with an AD8 score ≥2 were considered to have possible cognitive impairment, according to previous meta-analyses.^42,43^ The date of the first health checkup with cognitive function tested during the recruitment period was set as the baseline date for each individual. A 2-year “look-back” period from 2016 to 2017 was set to extract disease history. We excluded individuals with dementia diagnosis or dementia-related medication dispensation (donepezil, rivastigmine, galantamine, memantine) before baseline (*n* = 724), individuals with suspected cognitive impairment at baseline (*n* = 4 084), who left the city without any follow-up records (*n* = 3 565), without any inpatient or outpatient visit during study period (*n* = 4 967), with data missing on covariates (*n* = 998, missing data level <1%), died or diagnosed with dementia within 6 months following baseline date (*n* = 392). Eventually, 176 150 eligible participants were included in the final analyses (Fig. 2).

Participants were then split into the osteoporosis group and the control group, based on whether they had a prior osteoporosis diagnosis at baseline. The osteoporosis group consisted of patients with a previous osteoporosis diagnosis in inpatient or outpatient encounters. International Classification of Diseases 10th Revision (ICD-10) codes M80 and M81 from the primary or secondary discharge diagnoses, and a combination of ICD-10 codes and disease terminologies in Chinese in the listed outpatient diagnoses were used to extract osteoporosis diagnoses. The control group comprised older adults without any inpatient or outpatient diagnosis of osteoporosis recorded. Additionally, we also classified osteoporosis patients according to different clinical characteristics, including inpatient or outpatient settings, osteoporotic fracture history, and osteoporosis medication use. History of osteoporotic fracture, including fracture of the hip, pelvis, wrist, spine, or proximal humerus without any coding for high-energy trauma (a fall from one level to another, a transport accident, or exposure to mechanical forces) was identified via inpatient and outpatient diagnoses in accordance with prior literature (Table S12 for code lists).^44,45^ A validation study was conducted to assess the reliability of osteoporotic fracture ascertainment (see details in Supplementary Methods). Osteoporosis medication use (including calcium supplements, vitamin D, bisphosphonates, calcitonin, denosumab, estrogen, or others) was identified using drug-related terminologies in Chinese from inpatient and outpatient prescription records, supplemented by self-reported medications during health checkups.

### Outcome ascertainment and follow-up

Incident all-cause dementia was the main outcome of interest, collected from multiple sources, inclusive of outpatient and inpatient medical records and death registration records. We used ICD-10 codes in all listed discharge diagnoses or death causes, and a combination of ICD-10 codes and disease terminologies in Chinese in all listed outpatient diagnoses to identify dementia (Table S12). Incident AD and VD were treated as secondary outcomes because overlapping and evolving symptoms may lead to ambiguity in the diagnosis of dementia type.^12,46^ Follow-up for incident dementia began at the baseline date and continued until an earliest dementia diagnosis, death, or end of follow-up (June 30th, 2023), whichever came first. For those moved away, the last recorded visit date on the platform was used.

### Assessment of covariates

Sociodemographic characteristics, including date of birth, sex, ethnicity, education, marital status, and household registration, were retrieved from the resident health records. Education level was classified into 2 groups (junior high school or below, or senior high school or above). Household registration was divided into local (i.e., Shenzhen) or non-local. Information on lifestyles, including smoking status (never, former, or current smoker), and exercise frequency (<1 or ≥1 time per week), and physical examinations, including height, weight, and blood pressure, were assessed by primary care staff and recorded in the geriatric health management records. Blood pressure in both arms was measured, and systolic blood pressure and diastolic blood pressure were calculated as the mean of all available measurements. BMI was calculated as weight in kilograms divided by height in meters squared and categorized based on appropriate cutoff points for Chinese adults (<24, 24–28, and ≥28 kg/m^2^). Medical history was obtained through multiple sources, including self-reported disease history from the resident records, disease reported or detected during the health checkups, and diagnostic codes recorded in the outpatient and inpatient medical records (Table S12).

### Statistical analysis

Baseline characteristics of participants were presented as mean ± SD or median (IQR) for continuous variables or as frequency (percentage) for categorical variables. Cumulative incidence plots were used to compare the absolute risk of developing dementia over time and univariate Poisson regression models with the log of follow-up time as an offset variable were used to estimate the incidence rates and 95% CIs of subsequent dementia. Further, HRs and 95% CIs of dementia events associated with osteoporosis were calculated by multivariate Cox proportional hazard regression models. The proportional hazards assumption was assessed for all Cox models using Schoenfeld residual tests and no evidence of violation was detected. To minimize potential confounding bias, three models with sequentially greater degrees of adjustment were performed. Confounders were selected based on data availability and prior evidence of their potential associations with both osteoporosis and dementia. Model 1 included age at baseline, sex, education, marital status, and household registration. Model 2 additionally included smoking status, physical activity, BMI category, and systolic blood pressure. Model 3 further adjusted for major chronic conditions that have been reported to be associated with bone health and dementia, including hypertension, diabetes, dyslipidemia, stroke, cancer, chronic kidney disease, and depression.^3,47–51^ Subgroup analyses by age groups (<80 vs. ≥80 years), sex, BMI category, and presence or absence of baseline comorbidities were further conducted for the primary outcomes only, and multiplicative interactions were evaluated using the likelihood ratio test. In exploratory analyses, to reflect the severity of osteoporosis and to explore the potential impact of osteoporosis treatment, we further evaluated the risk of incident dementia among osteoporosis patients according to inpatient or outpatient settings, osteoporotic fracture history, and medication use.

To enhance the reliability of the results, we repeated the above analyses using a propensity score-matched cohort design. Specifically, PSM was employed to match each osteoporosis patient to up to 5 controls without replacement using the nearest neighbor matching algorithm with a caliper of 0.1. The propensity scores were calculated by logistic regression, accounting for the covariates in the above fully adjusted models. Standardized mean difference before and after PSM was computed and visualized to assess the covariate balance. Cox models with a robust variance estimator that accounts for clustering within matched pairs were used in the matched cohort analysis.^52^

We performed extensive sensitivity analyses to assess the stability and robustness of our findings. First, we performed subdistribution hazard (Fine-Gray) competing risk regression models treating death without dementia as the competing event. Second, a more stringent definition of dementia was used by requiring at least 2 outpatient records, 1 inpatient record, or 1 death record for dementia. Third, to reduce the risk of ascertainment bias, the number of outpatient and inpatient visits 12 months before baseline was additionally adjusted, and we further restricted participants in the control group to those with at least 1 outpatient or inpatient record before baseline. Fourth, we excluded incident dementia cases diagnosed within 1 year after the baseline to reduce the possibility of reverse causation. Fifth, individuals with benzodiazepine or anticholinergic drugs use within 3 months before baseline were further excluded, as these drugs have been linked with dementia.^53,54^ Sixth, to reduce potential misclassification bias, the control group was further restricted to a subset of older adults who had undergone BMD screening at baseline using quantitative ultrasound and were not identified as having osteoporosis. Furthermore, we further excluded individuals with a history of stroke or thyroid disease, adjusted for the use of antihypertensive, anti-hyperglycemia, lipid-lowering, antiplatelet medications, and proton pump inhibitors, and calculated the E-value for the main results to estimate the minimum magnitude of unmeasured confounding needed to explain away the observed association.^55^ Finally, incident osteoporosis during the follow-up period was treated as a time-varying exposure.

All analyses were performed with R (version 4.1.2) and SAS (version 9.4) softwares. Two-sided *P* < 0.05 was considered statistically significant.

## Supplementary information

Supplementary materials

## Data Availability

The raw data used in this study are subject to government policy restrictions and therefore cannot be made publicly available.

## Code availability

The codes used during the study are available from the corresponding author upon reasonable request.
