# Supplementary material for: Osteoporosis and risk of dementia among older adults: a population‑based cohort study
Source: Bone Res. 2025 Dec 22;13:104. doi: 10.1038/s41413-025-00480-7 (PMC12719389; doi:10.1038/s41413-025-00480-7)
Supplement: Supplementary file 1 — Supplementary materials [file 41413_2025_480_MOESM1_ESM.docx]

**Supplementary materials**

Osteoporosis and Risk of Dementia among Older Adults: A Population‑Based Cohort Study

[Table S1. Inpatient or outpatient settings, osteoporotic fracture history, and osteoporosis medication use in the osteoporosis group 2](#_Toc206088504)

[Table S2. Incident rates and Hazard ratios for all-cause dementia, AD, and VD in osteoporosis patients by inpatient or outpatient settings 3](#_Toc206088505)

[Table S3. Incident rates and Hazard ratios for all-cause dementia, AD, and VD in osteoporosis patients by osteoporosis medication use 5](#_Toc206088506)

[Table S4. Incident rates and Hazard ratios for all-cause dementia, AD, and VD for the osteoporosis patients and control groups using Fine-Gray models with death without dementia as competing events 7](#_Toc206088507)

[Table S5. Incident rates and Hazard ratios for all-cause dementia, AD, and VD for the osteoporosis patients and control groups when at least 1 inpatient record, 1 death record, or 2 outpatient records were required for dementia diagnosis 8](#_Toc206088508)

[Table S6. Incident rates and Hazard ratios for all-cause dementia, AD, and VD for the osteoporosis patients and control groups when the number of inpatient or outpatient clinical visits within 12 months before baseline was further adjusted 9](#_Toc206088509)

[Table S7. Incident rates and Hazard ratios for all-cause dementia, AD, and VD for the osteoporosis patients and control groups when further restricted participants to those with at least 1 inpatient or outpatient clinical visit before baseline 10](#_Toc206088510)

[Table S8. Incident rates and Hazard ratios for all-cause dementia, AD, and VD for the osteoporosis patients and control groups when incident dementia cases that occurred within 1 year after baseline were excluded 11](#_Toc206088511)

[Table S9. Incident rates and Hazard ratios for all-cause dementia, AD, and VD for the osteoporosis patients and control groups when individuals with benzodiazepine or anticholinergic drugs use within 3 months before baseline were excluded 12](#_Toc206088512)

[Table S10. Incident rates and Hazard ratios for all-cause dementia, AD, and VD for the osteoporosis patients and control groups in other sensitivity analyses 13](#_Toc206088513)

[Table S11. Incident rates and Hazard ratios for all-cause dementia, AD, and VD for the osteoporosis patients and control groups when incident osteoporosis during follow-up was treated as a time-varying exposure 15](#_Toc206088514)

[Table S12. ICD-10 codes used in the present study 16](#_Toc206088515)

[Figure S1. Absolute standardized mean difference before and after propensity score matching 17](#_Toc206088516)

[Figure S2. Cumulative incidence of all-cause dementia, AD, and VD for the osteoporosis and control groups 18](#_Toc206088517)

[Figure S3. Association between osteoporosis and all-cause dementia by subgroups 19](#_Toc206088518)

[Figure S4. The E-value analysis based on multivariable adjusted model for the observed association between osteoporosis and dementia 20](#_Toc206088519)

[Supplementary Methods 21](#_Toc206088520)

### Table S1. Inpatient or outpatient settings, osteoporotic fracture history, and osteoporosis medication use in the osteoporosis group

| Clinical characteristics | Osteoporosis group (n = 9605) |
| --- | --- |
| Inpatient or outpatient settings^*^ |  |
| Inpatient | 3361 (35.0) |
| Outpatient | 6244 (65.0) |
| Osteoporotic fractures | 1772 (18.4) |
| Osteoporotic medications | 3625 (37.7) |
| Osteoporotic medication categories |  |
| Calcium supplements | 2533 (26.4) |
| Vitamin D (%) | 3294 (34.3) |
| Bisphosphonates (%) | 535 (5.6) |
| Calcitonin (%) | 336 (3.5) |
| Other^&^ | 427 (4.4) |

^*^ Patients with an inpatient diagnosis of osteoporosis were excluded from the group of osteoporosis outpatients.

^&^ Other drugs include denosumab, estrogen, raloxifene, teriparatide and menatetrenone.

### Table S2. Incident rates and Hazard ratios for all-cause dementia, AD, and VD in osteoporosis patients by inpatient or outpatient settings

| **Outcome** | **N** | **No. of events** | **Person-years** | **IR (95% CI), per 1,000 person-years** | **Model 1** | | **Model 2** | | **Model 3** | |
| --- | --- | --- | --- | --- | --- | --- | --- | --- | --- | --- |
|  |  |  |  |  | HR (95% CI) | *P* | HR (95% CI) | *P* | HR (95% CI) | *P* |
| **Full cohort analysis** |  |  |  |  |  |  |  |  |  |  |
| **Dementia** |  |  |  |  |  |  |  |  |  |  |
| Control group | 166545 | 1185 | 481609.0 | 2.46 (2.32-2.60) | ref |  | ref |  | ref |  |
| Osteoporosis outpatients | 6244 | 82 | 14683.0 | 5.58 (4.44-6.93) | 1.87 (1.49-2.35) | <0.001 | 1.83 (1.46-2.30) | <0.001 | 1.66 (1.32-2.09) | <0.001 |
| Osteoporosis inpatients | 3361 | 100 | 9130.9 | 10.95 (8.91-13.32) | 2.52 (2.04-3.11) | <0.001 | 2.44 (1.97-3.01) | <0.001 | 1.94 (1.56-2.40) | <0.001 |
| **AD** |  |  |  |  |  |  |  |  |  |  |
| Control group | 166545 | 543 | 482491.3 | 1.13 (1.03-1.22) | ref |  | ref |  | ref |  |
| Osteoporosis outpatients | 6244 | 32 | 14753.0 | 2.17 (1.48-3.06) | 1.61 (1.12-2.31) | 0.010 | 1.57 (1.09-2.25) | 0.015 | 1.43 (0.99-2.06) | 0.055 |
| Osteoporosis inpatients | 3361 | 42 | 9211.7 | 4.56 (3.29-6.16) | 2.20 (1.59-3.04) | <0.001 | 2.12 (1.54-2.93) | <0.001 | 1.66 (1.20-2.31) | 0.002 |
| **VD** |  |  |  |  |  |  |  |  |  |  |
| Control group | 166545 | 252 | 482825.2 | 0.52 (0.46-0.59) | ref |  | ref |  | ref |  |
| Osteoporosis outpatients | 6244 | 14 | 14771.0 | 0.95 (0.52-1.59) | 1.64 (0.95-2.82) | 0.077 | 1.62 (0.94-2.80) | 0.083 | 1.33 (0.77-2.30) | 0.311 |
| Osteoporosis inpatients | 3361 | 32 | 9221.5 | 3.47 (2.37-4.90) | 4.57 (3.11-6.72) | <0.001 | 4.46 (3.03-6.57) | <0.001 | 2.94 (1.98-4.36) | <0.001 |
| **Matched cohort analysis** |  |  |  |  |  |  |  |  |  |  |
| **Dementia** |  |  |  |  |  |  |  |  |  |  |
| Control group | 43913 | 526 | 130015.3 | 4.05 (3.71-4.41) | ref |  | ref |  | ref |  |
| Osteoporosis outpatients | 6211 | 80 | 14608.6 | 5.48 (4.34-6.82) | 1.54 (1.20-1.96) | 0.001 | 1.53 (1.20-1.95) | 0.001 | 1.61 (1.26-2.05) | <0.001 |
| Osteoporosis inpatients | 3319 | 95 | 9034.8 | 10.51 (8.51-12.85) | 2.16 (1.72-2.72) | <0.001 | 2.13 (1.69-2.68) | <0.001 | 1.98 (1.57-2.49) | <0.001 |
| **AD** |  |  |  |  |  |  |  |  |  |  |
| Control group | 43913 | 250 | 130401.1 | 1.92 (1.69-2.17) | ref |  | ref |  | ref |  |
| Osteoporosis outpatients | 6211 | 32 | 14678.1 | 2.18 (1.49-3.08) | 1.36 (0.93-1.99) | 0.108 | 1.35 (0.93-1.97) | 0.119 | 1.43 (0.98-2.08) | 0.064 |
| Osteoporosis inpatients | 3319 | 40 | 9107.2 | 4.39 (3.14-5.98) | 1.94 (1.37-2.73) | <0.001 | 1.90 (1.35-2.68) | <0.001 | 1.75 (1.24-2.48) | 0.002 |
| **VD** |  |  |  |  |  |  |  |  |  |  |
| Control group | 43913 | 118 | 130558.7 | 0.90 (0.75-1.08) | ref |  | ref |  | ref |  |
| Osteoporosis outpatients | 6211 | 14 | 14696.1 | 0.95 (0.52-1.60) | 1.12 (0.63-1.97) | 0.704 | 1.11 (0.63-1.96) | 0.721 | 1.19 (0.67-2.10) | 0.552 |
| Osteoporosis inpatients | 3319 | 31 | 9116.2 | 3.40 (2.31-4.83) | 3.09 (2.04-4.67) | <0.001 | 3.05 (2.02-4.60) | <0.001 | 2.69 (1.77-4.09) | <0.001 |

Abbreviations: AD, Alzheimer's disease; VD, vascular dementia; IR, incidence rate; CI, confidence interval; HR, hazard ratio.

Patients with an inpatient diagnosis of osteoporosis were excluded from the group of osteoporosis outpatients.

Model 1: adjusted for age at baseline, sex, education, marital status, and household registration;

Model 2: Model 1 plus smoking, regular exercise, body mass index category, and systolic blood pressure;

Model 3: Model 2 plus disease history, including hypertension, diabetes, dyslipidemia, stroke, cancer, chronic kidney disease, and depression.

### Table S3. Incident rates and Hazard ratios for all-cause dementia, AD, and VD in osteoporosis patients by osteoporosis medication use

| **Outcome** | **N** | **No. of events** | **Person-years** | **IR (95% CI), per 1,000 person-years** | **Model 1** | | **Model 2** | | **Model 3** | |
| --- | --- | --- | --- | --- | --- | --- | --- | --- | --- | --- |
|  |  |  |  |  | HR (95% CI) | *P* | HR (95% CI) | *P* | HR (95% CI) | *P* |
| **Full cohort analysis** |  |  |  |  |  |  |  |  |  |  |
| **Dementia** |  |  |  |  |  |  |  |  |  |  |
| Control group | 166545 | 1185 | 481609.0 | 2.46 (2.32-2.60) | ref |  | ref |  | ref |  |
| Without osteoporotic medications | 5980 | 155 | 16637.6 | 9.32 (7.91-10.90) | 2.34 (1.97-2.77) | <0.001 | 2.27 (1.91-2.70) | <0.001 | 1.94 (1.63-2.31) | <0.001 |
| With osteoporotic medications | 3625 | 27 | 7176.3 | 3.76 (2.48-5.47) | 1.56 (1.06-2.29) | 0.025 | 1.52 (1.03-2.24) | 0.033 | 1.25 (0.84-1.84) | 0.266 |
| **AD** |  |  |  |  |  |  |  |  |  |  |
| Control group | 166545 | 543 | 482491.3 | 1.13 (1.03-1.22) | ref |  | ref |  | ref |  |
| Without osteoporotic medications | 5980 | 64 | 16772.6 | 3.82 (2.94-4.87) | 2.01 (1.54-2.62) | <0.001 | 1.94 (1.49-2.53) | <0.001 | 1.64 (1.25-2.15) | <0.001 |
| With osteoporotic medications | 3625 | 10 | 7192.1 | 1.39 (0.67-2.56) | 1.39 (0.74-2.61) | 0.312 | 1.36 (0.72-2.57) | 0.340 | 1.12 (0.59-2.13) | 0.721 |
| **VD** |  |  |  |  |  |  |  |  |  |  |
| Control group | 166545 | 252 | 482825.2 | 0.52 (0.46-0.59) | ref |  | ref |  | ref |  |
| Without osteoporotic medications | 5980 | 42 | 16799.1 | 2.50 (1.80-3.38) | 3.35 (2.38-4.70) | <0.001 | 3.30 (2.35-4.63) | <0.001 | 2.44 (1.72-3.44) | <0.001 |
| With osteoporotic medications | 3625 | 4 | 7193.5 | 0.56 (0.15-1.42) | 1.26 (0.46-3.41) | 0.655 | 1.23 (0.45-3.35) | 0.681 | 0.89 (0.32-2.42) | 0.812 |
| **Matched cohort analysis** |  |  |  |  |  |  |  |  |  |  |
| **Dementia** |  |  |  |  |  |  |  |  |  |  |
| Control group | 43913 | 526 | 130015.3 | 4.05 (3.71-4.41) | ref |  | ref |  | ref |  |
| Without osteoporotic medications | 5940 | 149 | 16534.3 | 9.01 (7.62-10.58) | 1.97 (1.63-2.38) | <0.001 | 1.94 (1.61-2.35) | <0.001 | 1.93 (1.60-2.34) | <0.001 |
| With osteoporotic medications | 3590 | 26 | 7109.1 | 3.66 (2.39-5.36) | 1.29 (0.86-1.92) | 0.219 | 1.28 (0.85-1.91) | 0.232 | 1.24 (0.83-1.86) | 0.297 |
| **AD** |  |  |  |  |  |  |  |  |  |  |
| Control group | 43913 | 250 | 130401.1 | 1.92 (1.69-2.17) | ref |  | ref |  | ref |  |
| Without osteoporotic medications | 5940 | 62 | 16660.5 | 3.72 (2.85-4.77) | 1.74 (1.31-2.31) | <0.001 | 1.72 (1.29-2.28) | <0.001 | 1.70 (1.28-2.25) | <0.001 |
| With osteoporotic medications | 3590 | 10 | 7124.8 | 1.40 (0.67-2.58) | 1.17 (0.61-2.23) | 0.634 | 1.15 (0.60-2.20) | 0.665 | 1.14 (0.60-2.18) | 0.694 |
| **VD** |  |  |  |  |  |  |  |  |  |  |
| Control group | 43913 | 118 | 130558.7 | 0.90 (0.75-1.08) | ref |  | ref |  | ref |  |
| Without osteoporotic medications | 5940 | 41 | 16686.1 | 2.46 (1.76-3.33) | 2.29 (1.60-3.30) | <0.001 | 2.28 (1.59-3.27) | <0.001 | 2.22 (1.54-3.21) | <0.001 |
| With osteoporotic medications | 3590 | 4 | 7126.2 | 0.56 (0.15-1.44) | 0.85 (0.31-2.31) | 0.746 | 0.83 (0.30-2.28) | 0.719 | 0.82 (0.30-2.25) | 0.695 |

Abbreviations: AD, Alzheimer's disease; VD, vascular dementia; IR, incidence rate; CI, confidence interval; HR, hazard ratio.

Osteoporosis medications include calcium and vitamin D supplements, bisphosphonates (alendronate, ibandronate, risedronate, zoledronic acid), calcitonin, denosumab, estrogen, raloxifene, teriparatide, and menatetrenone. Due to the low treatment rate of osteoporosis, we are unable to provide estimates specifically for each individual medication.

Model 1: adjusted for age at baseline, sex, education, marital status, and household registration;

Model 2: Model 1 plus smoking, regular exercise, body mass index category, and systolic blood pressure;

Model 3: Model 2 plus disease history, including hypertension, diabetes, dyslipidemia, stroke, cancer, chronic kidney disease, and depression.

### Table S4. Incident rates and Hazard ratios for all-cause dementia, AD, and VD for the osteoporosis patients and control groups using Fine-Gray models with death without dementia as competing events

| **Outcome** | **N** | **No. of events** | **No. of competing deaths** | **Person-years** | **Model 1** | | **Model 2** | | **Model 3** | |
| --- | --- | --- | --- | --- | --- | --- | --- | --- | --- | --- |
|  |  |  |  |  | HR (95% CI) | *P* | HR (95% CI) | *P* | HR (95% CI) | *P* |
| **Full cohort analysis** |  |  |  |  |  |  |  |  |  |  |
| **Dementia** |  |  |  |  |  |  |  |  |  |  |
| Control group | 166545 | 1185 | 3183 | 481609.0 | ref |  | ref |  | ref |  |
| Osteoporosis group | 9605 | 182 | 278 | 23813.9 | 2.11 (1.79-2.48) | <0.001 | 2.05 (1.74-2.42) | <0.001 | 1.76 (1.48-2.08) | <0.001 |
| **AD** |  |  |  |  |  |  |  |  |  |  |
| Control group | 166545 | 543 | 3301 | 482491.3 | ref |  | ref |  | ref |  |
| Osteoporosis group | 9605 | 74 | 291 | 23964.7 | 1.82 (1.42-2.35) | <0.001 | 1.77 (1.37-2.29) | <0.001 | 1.51 (1.16-1.96) | 0.002 |
| **VD** |  |  |  |  |  |  |  |  |  |  |
| Control group | 166545 | 252 | 3357 | 482825.2 | ref |  | ref |  | ref |  |
| Osteoporosis group | 9605 | 46 | 297 | 23992.5 | 2.84 (2.04-3.96) | <0.001 | 2.80 (2.01-3.90) | <0.001 | 2.06 (1.46-2.91) | <0.001 |
| **Matched cohort analysis** |  |  |  |  |  |  |  |  |  |  |
| **Dementia** |  |  |  |  |  |  |  |  |  |  |
| Control group | 43913 | 526 | 1280 | 130015.3 | ref |  | ref |  | ref |  |
| Osteoporosis group | 9530 | 175 | 264 | 23643.4 | 1.79 (1.51-2.12) | <0.001 | 1.77 (1.50-2.10) | <0.001 | 1.75 (1.48-2.07) | <0.001 |
| **AD** |  |  |  |  |  |  |  |  |  |  |
| Control group | 43913 | 250 | 1342 | 130401.1 | ref |  | ref |  | ref |  |
| Osteoporosis group | 9530 | 72 | 277 | 23785.3 | 1.59 (1.23-2.07) | <0.001 | 1.57 (1.21-2.04) | <0.001 | 1.55 (1.19-2.01) | 0.001 |
| **VD** |  |  |  |  |  |  |  |  |  |  |
| Control group | 43913 | 118 | 1364 | 130558.7 | ref |  | ref |  | ref |  |
| Osteoporosis group | 9530 | 45 | 283 | 23812.3 | 1.96 (1.39-2.75) | <0.001 | 1.94 (1.38-2.72) | <0.001 | 1.88 (1.34-2.65) | <0.001 |

Abbreviations: AD, Alzheimer's disease; VD, vascular dementia; IR, incidence rate; CIs, confidence interval; HR, hazard ratio

Model 1: adjusted for age at baseline, sex, education, marital status, and household registration;

Model 2: Model 1 plus smoking, regular exercise, body mass index category, and systolic blood pressure;

Model 3: Model 2 plus disease history, including hypertension, diabetes, dyslipidemia, stroke, cancer, chronic kidney disease, and depression.

### Table S5. Incident rates and Hazard ratios for all-cause dementia, AD, and VD for the osteoporosis patients and control groups when at least 1 inpatient record, 1 death record, or 2 outpatient records were required for dementia diagnosis

| **Outcome** | **N** | **No. of events** | **Person-years** | **IR (95% CI), per 1,000 person-years** | **Model 1** | | **Model 2** | | **Model 3** | |
| --- | --- | --- | --- | --- | --- | --- | --- | --- | --- | --- |
|  |  |  |  |  | HR (95% CI) | *P* | HR (95% CI) | *P* | HR (95% CI) | *P* |
| **Full cohort analysis** |  |  |  |  |  |  |  |  |  |  |
| **Dementia** |  |  |  |  |  |  |  |  |  |  |
| Control group | 166545 | 967 | 481865.0 | 2.01 (1.88-2.14) | ref |  | ref |  | ref |  |
| Osteoporosis group | 9605 | 143 | 23854.8 | 5.99 (5.05-7.06) | 2.02 (1.69-2.43) | <0.001 | 1.96 (1.64-2.36) | <0.001 | 1.67 (1.38-2.01) | <0.001 |
| **AD** |  |  |  |  |  |  |  |  |  |  |
| Control group | 166545 | 495 | 482535.6 | 1.03 (0.94-1.12) | ref |  | ref |  | ref |  |
| Osteoporosis group | 9605 | 66 | 23975.7 | 2.75 (2.13-3.50) | 1.81 (1.39-2.35) | <0.001 | 1.75 (1.34-2.28) | <0.001 | 1.50 (1.15-1.97) | 0.003 |
| **VD** |  |  |  |  |  |  |  |  |  |  |
| Control group | 166545 | 199 | 482875.2 | 0.41 (0.36-0.47) | ref |  | ref |  | ref |  |
| Osteoporosis group | 9605 | 40 | 23998.6 | 1.67 (1.19-2.27) | 3.12 (2.19-4.45) | <0.001 | 3.06 (2.14-4.37) | <0.001 | 2.21 (1.53-3.18) | <0.001 |
| **Matched cohort analysis** |  |  |  |  |  |  |  |  |  |  |
| **Dementia** |  |  |  |  |  |  |  |  |  |  |
| Control group | 43913 | 447 | 130109.8 | 3.44 (3.12-3.77) | ref |  | ref |  | ref |  |
| Osteoporosis group | 9530 | 138 | 23681.2 | 5.83 (4.90-6.88) | 1.71 (1.40-2.09) | <0.001 | 1.69 (1.39-2.07) | <0.001 | 1.68 (1.37-2.05) | <0.001 |
| **AD** |  |  |  |  |  |  |  |  |  |  |
| Control group | 43913 | 229 | 130416.3 | 1.76 (1.54-2.00) | ref |  | ref |  | ref |  |
| Osteoporosis group | 9530 | 64 | 23796.3 | 2.69 (2.07-3.43) | 1.59 (1.19-2.12) | 0.002 | 1.56 (1.17-2.09) | 0.003 | 1.54 (1.16-2.06) | 0.003 |
| **VD** |  |  |  |  |  |  |  |  |  |  |
| Control group | 43913 | 97 | 130572.6 | 0.74 (0.60-0.91) | ref |  | ref |  | ref |  |
| Osteoporosis group | 9530 | 39 | 23818.4 | 1.64 (1.16-2.24) | 2.12 (1.44-3.11) | <0.001 | 2.10 (1.43-3.08) | <0.001 | 2.04 (1.38-3.01) | <0.001 |

Abbreviations: AD, Alzheimer's disease; VD, vascular dementia; IR, incidence rate; CIs, confidence interval; HR, hazard ratio

Model 1: adjusted for age at baseline, sex, education, marital status and household registration;

Model 2: Model 1 plus smoking, regular exercise, body mass index category, and systolic blood pressure;

Model 3: Model 2 plus disease history, including hypertension, diabetes, dyslipidemia, stroke, cancer, chronic kidney disease, and depression.

### Table S6. Incident rates and Hazard ratios for all-cause dementia, AD, and VD for the osteoporosis patients and control groups when the number of inpatient or outpatient clinical visits within 12 months before baseline was further adjusted

| **Outcome** | **N** | **No. of events** | **Person-years** | **IR (95% CI), per 1,000 person-years** | **HR (95% CI)** | ***P*** |
| --- | --- | --- | --- | --- | --- | --- |
| **Full cohort analysis** |  |  |  |  |  |  |
| **Dementia** |  |  |  |  |  |  |
| Control group | 166545 | 1185 | 481609.0 | 2.46 (2.32-2.60) | ref |  |
| Osteoporosis group | 9605 | 182 | 23813.9 | 7.64 (6.57-8.84) | 1.72 (1.45-2.04) | <0.001 |
| **AD** |  |  |  |  |  |  |
| Control group | 166545 | 543 | 482491.3 | 1.13 (1.03-1.22) | ref |  |
| Osteoporosis group | 9605 | 74 | 23964.7 | 3.09 (2.42-3.88) | 1.50 (1.15-1.95) | 0.002 |
| **VD** |  |  |  |  |  |  |
| Control group | 166545 | 252 | 482825.2 | 0.52 (0.46-0.59) | ref |  |
| Osteoporosis group | 9605 | 46 | 23992.5 | 1.92 (1.40-2.56) | 2.19 (1.55-3.10) | <0.001 |
| **Matched cohort analysis** |  |  |  |  |  |  |
| **Dementia** |  |  |  |  |  |  |
| Control group | 43913 | 526 | 130015.3 | 4.05 (3.71-4.41) | ref |  |
| Osteoporosis group | 9530 | 175 | 23643.4 | 7.40 (6.35-8.58) | 1.74 (1.43-2.11) | <0.001 |
| **AD** |  |  |  |  |  |  |
| Control group | 43913 | 250 | 130401.1 | 1.92 (1.69-2.17) | ref |  |
| Osteoporosis group | 9530 | 72 | 23785.3 | 3.03 (2.37-3.81) | 1.57 (1.17-2.09) | 0.002 |
| **VD** |  |  |  |  |  |  |
| Control group | 43913 | 118 | 130558.7 | 0.90 (0.75-1.08) | ref |  |
| Osteoporosis group | 9530 | 45 | 23812.3 | 1.89 (1.38-2.53) | 2.08 (1.42-3.05) | <0.001 |

Abbreviations: AD, Alzheimer's disease; VD, vascular dementia; IR, incidence rate; CIs, confidence interval; HR, hazard ratio

Model were adjusted for age at baseline, sex, education, marital status household registration, smoking, regular exercise, body mass index category, systolic blood pressure, disease history (including hypertension, diabetes, dyslipidemia, stroke, cancer, chronic kidney disease, and depression) and the number of inpatient or outpatient clinical visits within 12 months before baseline.

### Table S7. Incident rates and Hazard ratios for all-cause dementia, AD, and VD for the osteoporosis patients and control groups when further restricted participants to those with at least 1 inpatient or outpatient clinical visit before baseline

| **Outcome** | **N** | **No. of events** | **Person-years** | **IR (95% CI), per 1,000 person-years** | **Model 1** | | **Model 2** | | **Model 3** | |
| --- | --- | --- | --- | --- | --- | --- | --- | --- | --- | --- |
|  |  |  |  |  | HR (95% CI) | *P* | HR (95% CI) | *P* | HR (95% CI) | *P* |
| **Full cohort analysis** |  |  |  |  |  |  |  |  |  |  |
| **Dementia** |  |  |  |  |  |  |  |  |  |  |
| Control group | 127612 | 796 | 316060.3 | 2.52 (2.35-2.70) | ref |  | ref |  | ref |  |
| Osteoporosis group | 9542 | 181 | 23576.9 | 7.68 (6.60-8.88) | 1.94 (1.64-2.29) | <0.001 | 1.91 (1.62-2.26) | <0.001 | 1.74 (1.46-2.06) | <0.001 |
| **AD** |  |  |  |  |  |  |  |  |  |  |
| Control group | 127612 | 364 | 316625.3 | 1.15 (1.03-1.27) | ref |  | ref |  | ref |  |
| Osteoporosis group | 9542 | 74 | 23725.6 | 3.12 (2.45-3.92) | 1.67 (1.29-2.16) | <0.001 | 1.64 (1.26-2.12) | <0.001 | 1.49 (1.15-1.94) | 0.003 |
| **VD** |  |  |  |  |  |  |  |  |  |  |
| Control group | 127612 | 175 | 316822.3 | 0.55 (0.47-0.64) | ref |  | ref |  | ref |  |
| Osteoporosis group | 9542 | 45 | 23755.5 | 1.89 (1.38-2.53) | 2.47 (1.75-3.47) | <0.001 | 2.44 (1.73-3.43) | <0.001 | 2.03 (1.44-2.87) | <0.001 |
| **Matched cohort analysis** |  |  |  |  |  |  |  |  |  |  |
| **Dementia** |  |  |  |  |  |  |  |  |  |  |
| Control group | 35743 | 392 | 93928.6 | 4.17 (3.77-4.61) | ref |  | ref |  | ref |  |
| Osteoporosis group | 9467 | 174 | 23406.4 | 7.43 (6.37-8.62) | 1.66 (1.37-2.00) | <0.001 | 1.65 (1.37-2.00) | <0.001 | 1.75 (1.45-2.11) | <0.001 |
| **AD** |  |  |  |  |  |  |  |  |  |  |
| Control group | 35743 | 185 | 94214.1 | 1.96 (1.69-2.27) | ref |  | ref |  | ref |  |
| Osteoporosis group | 9467 | 72 | 23546.2 | 3.06 (2.39-3.85) | 1.48 (1.12-1.96) | 0.007 | 1.47 (1.11-1.95) | 0.007 | 1.55 (1.17-2.06) | 0.002 |
| **VD** |  |  |  |  |  |  |  |  |  |  |
| Control group | 35743 | 90 | 94310.4 | 0.95 (0.77-1.17) | ref |  | ref |  | ref |  |
| Osteoporosis group | 9467 | 44 | 23575.2 | 1.87 (1.36-2.51) | 1.74 (1.20-2.53) | 0.003 | 1.73 (1.19-2.50) | 0.004 | 1.89 (1.30-2.74) | 0.001 |

Abbreviations: AD, Alzheimer's disease; VD, vascular dementia; IR, incidence rate; CIs, confidence interval; HR, hazard ratio

Model 1: adjusted for age at baseline, sex, education, marital status, and household registration;

Model 2: Model 1 plus smoking, regular exercise, body mass index category, and systolic blood pressure;

Model 3: Model 2 plus disease history, including hypertension, diabetes, dyslipidemia, stroke, cancer, chronic kidney disease, and depression.

### Table S8. Incident rates and Hazard ratios for all-cause dementia, AD, and VD for the osteoporosis patients and control groups when incident dementia cases that occurred within 1 year after baseline were excluded

| **Outcome** | **N** | **No. of events** | **Person-years** | **IR (95% CI),** **per 1,000 person-years** | **Model 1** | | **Model 2** | | **Model 3** | |
| --- | --- | --- | --- | --- | --- | --- | --- | --- | --- | --- |
|  |  |  |  |  | HR (95% CI) | *P* | HR (95% CI) | *P* | HR (95% CI) | *P* |
| **Full cohort analysis** |  |  |  |  |  |  |  |  |  |  |
| **Dementia** |  |  |  |  |  |  |  |  |  |  |
| Control group | 166420 | 1060 | 481517.1 | 2.20 (2.07-2.34) | ref |  | ref |  | ref |  |
| Osteoporosis group | 9573 | 150 | 23789.7 | 6.31 (5.34-7.40) | 2.12 (1.78-2.54) | <0.001 | 2.07 (1.73-2.47) | <0.001 | 1.77 (1.47-2.12) | <0.001 |
| **AD** |  |  |  |  |  |  |  |  |  |  |
| Control group | 166420 | 483 | 482265.2 | 1.00 (0.91-1.09) | ref |  | ref |  | ref |  |
| Osteoporosis group | 9573 | 61 | 23908.3 | 2.55 (1.95-3.28) | 1.86 (1.41-2.45) | <0.001 | 1.81 (1.37-2.38) | <0.001 | 1.50 (1.14-1.99) | 0.004 |
| **VD** |  |  |  |  |  |  |  |  |  |  |
| Control group | 166420 | 218 | 482556.9 | 0.45 (0.39-0.52) | ref |  | ref |  | ref |  |
| Osteoporosis group | 9573 | 36 | 23937.9 | 1.50 (1.05-2.08) | 2.89 (2.00-4.17) | <0.001 | 2.84 (1.96-4.10) | <0.001 | 2.12 (1.45-3.08) | <0.001 |
| **Matched cohort analysis** |  |  |  |  |  |  |  |  |  |  |
| **Dementia** |  |  |  |  |  |  |  |  |  |  |
| Control group | 43724 | 461 | 129582.4 | 3.56 (3.24-3.90) | ref |  | ref |  | ref |  |
| Osteoporosis group | 9499 | 144 | 23620.1 | 6.10 (5.14-7.18) | 1.79 (1.46-2.18) | <0.001 | 1.77 (1.45-2.16) | <0.001 | 1.74 (1.43-2.13) | <0.001 |
| **AD** |  |  |  |  |  |  |  |  |  |  |
| Control group | 43724 | 217 | 129904.6 | 1.67 (1.46-1.91) | ref |  | ref |  | ref |  |
| Osteoporosis group | 9499 | 59 | 23731.5 | 2.49 (1.89-3.21) | 1.60 (1.19-2.16) | 0.002 | 1.58 (1.17-2.13) | 0.003 | 1.55 (1.15-2.10) | 0.004 |
| **VD** |  |  |  |  |  |  |  |  |  |  |
| Control group | 43724 | 97 | 130036.3 | 0.75 (0.60-0.91) | ref |  | ref |  | ref |  |
| Osteoporosis group | 9499 | 36 | 23758.6 | 1.52 (1.06-2.10) | 2.05 (1.38-3.06) | <0.001 | 2.04 (1.37-3.04) | <0.001 | 1.98 (1.32-2.96) | 0.001 |

Abbreviations: AD, Alzheimer's disease; VD, vascular dementia; IR, incidence rate; CIs, confidence interval; HR, hazard ratio

Model 1: adjusted for age at baseline, sex, education, marital status and household registration;

Model 2: Model 1 plus smoking, regular exercise, body mass index category, and systolic blood pressure;

Model 3: Model 2 plus disease history, including hypertension, diabetes, dyslipidemia, stroke, cancer, chronic kidney disease, and depression.

### Table S9. Incident rates and Hazard ratios for all-cause dementia, AD, and VD for the osteoporosis patients and control groups when individuals with benzodiazepine or anticholinergic drugs use within 3 months before baseline were excluded

| **Outcome** | **N** | **No. of events** | **Person-years** | **IR (95% CI), per 1,000 person-years** | **Model 1** | | | **Model 2** | | **Model 3** | |
| --- | --- | --- | --- | --- | --- | --- | --- | --- | --- | --- | --- |
|  |  |  |  |  | HR (95% CI) | *P* | HR (95% CI) | | *P* | HR (95% CI) | *P* |
| **Full cohort analysis** |  |  |  |  |  |  |  | |  |  |  |
| **Dementia** |  |  |  |  |  |  |  | |  |  |  |
| Control group | 163186 | 1163 | 474509 | 2.45 (2.31-2.60) | ref |  | ref | |  | ref |  |
| Osteoporosis group | 8905 | 171 | 22409.6 | 7.63 (6.53-8.86) | 2.18 (1.85-2.58) | <0.001 | 2.13 (1.80-2.51) | | <0.001 | 1.81 (1.53-2.14) | <0.001 |
| **AD** |  |  |  |  |  |  |  | |  |  |  |
| Control group | 163186 | 537 | 475379 | 1.13 (1.04-1.23) | ref |  | ref | |  | ref |  |
| Osteoporosis group | 8905 | 71 | 22554.8 | 3.15 (2.46-3.97) | 1.92 (1.49-2.48) | <0.001 | 1.86 (1.44-2.41) | | <0.001 | 1.57 (1.21-2.03) | 0.001 |
| **VD** |  |  |  |  |  |  |  | |  |  |  |
| Control group | 163186 | 250 | 475708.3 | 0.53 (0.46-0.59) | ref |  | ref | |  | ref |  |
| Osteoporosis group | 8905 | 44 | 22581.4 | 1.95 (1.42-2.62) | 2.97 (2.12-4.15) | <0.001 | 2.92 (2.09-4.09) | | <0.001 | 2.14 (1.52-3.02) | <0.001 |
| **Matched cohort analysis** |  |  |  |  |  |  |  | |  |  |  |
| **Dementia** |  |  |  |  |  |  |  | |  |  |  |
| Control group | 39710 | 467 | 118627.1 | 3.94 (3.59-4.31) | ref |  | ref | |  | ref |  |
| Osteoporosis group | 8848 | 164 | 22273.5 | 7.36 (6.28-8.58) | 1.89 (1.57-2.28) | <0.001 | 1.88 (1.56-2.26) | | <0.001 | 1.84 (1.53-2.22) | <0.001 |
| **AD** |  |  |  |  |  |  |  | |  |  |  |
| Control group | 39710 | 217 | 118969.7 | 1.82 (1.59-2.08) | ref |  | ref | |  | ref |  |
| Osteoporosis group | 8848 | 69 | 22409.8 | 3.08 (2.40-3.90) | 1.77 (1.34-2.34) | <0.001 | 1.75 (1.33-2.32) | | <0.001 | 1.72 (1.30-2.27) | <0.001 |
| **VD** |  |  |  |  |  |  |  | |  |  |  |
| Control group | 39710 | 100 | 119108.4 | 0.84 (0.68-1.02) | ref |  | ref | |  | ref |  |
| Osteoporosis group | 8848 | 43 | 22435.5 | 1.92 (1.39-2.58) | 2.22 (1.53-3.21) | <0.001 | 2.20 (1.52-3.18) | | <0.001 | 2.11 (1.45-3.07) | <0.001 |

Abbreviations: AD, Alzheimer's disease; VD, vascular dementia; IR, incidence rate; CIs, confidence interval; HR, hazard ratio

Benzodiazepines and anticholinergic drugs (including antihistamines, antidepressants, antiepileptics, antiparkinsonian agents, antipsychotics, and antispasmodics) that may affect cognitive function were chosen according to previous literature.

Model 1: adjusted for age at baseline, sex, education, marital status and household registration;

Model 2: Model 1 plus smoking, regular exercise, body mass index category, and systolic blood pressure;

Model 3: Model 2 plus disease history, including hypertension, diabetes, dyslipidemia, stroke, cancer, chronic kidney disease, and depression.

### Table S10. Incident rates and Hazard ratios for all-cause dementia, AD, and VD for the osteoporosis patients and control groups in other sensitivity analyses

| **Outcome** | **N** | **No. of events** | **Person-years** | **IR (95% CI), per 1,000 person-years** | **Model 1** | | **Model 2** | | **Model 3** | |
| --- | --- | --- | --- | --- | --- | --- | --- | --- | --- | --- |
|  |  |  |  |  | HR (95% CI) | *P* | HR (95% CI) | *P* | HR (95% CI) | *P* |
| **Excluding those with stroke disease history** | | | | | | | | | | |
| **Dementia** |  |  |  |  |  |  |  |  |  |  |
| Control group | 152702 | 925 | 444510.9 | 2.08 (1.95-2.22) | ref |  | ref |  | ref |  |
| Osteoporosis group | 7798 | 107 | 19479.7 | 5.49 (4.50-6.64) | 1.92 (1.56-2.36) | <0.001 | 1.87 (1.52-2.30) | <0.001 | 1.83 (1.49-2.26) | <0.001 |
| **AD** |  |  |  |  |  |  |  |  |  |  |
| Control group | 152702 | 416 | 445194.3 | 0.93 (0.85-1.03) | ref |  | ref |  | ref |  |
| Osteoporosis group | 7798 | 45 | 19566.2 | 2.30 (1.68-3.08) | 1.75 (1.28-2.40) | <0.001 | 1.70 (1.24-2.33) | 0.001 | 1.66 (1.21-2.29) | 0.002 |
| **VD** |  |  |  |  |  |  |  |  |  |  |
| Control group | 152702 | 166 | 445491.9 | 0.37 (0.32-0.43) | ref |  | ref |  | ref |  |
| Osteoporosis group | 7798 | 24 | 19595.8 | 1.22 (0.78-1.82) | 2.73 (1.75-4.26) | <0.001 | 2.69 (1.73-4.20) | <0.001 | 2.52 (1.61-3.96) | <0.001 |
| **Excluding those with thyroid disease history** | | | | | | | | | | |
| **Dementia** |  |  |  |  |  |  |  |  |  |  |
| Control group | 159980 | 1136 | 464464.2 | 2.45 (2.31-2.59) | ref |  | ref |  | ref |  |
| Osteoporosis group | 8147 | 148 | 20355.3 | 7.27 (6.15-8.54) | 2.06 (1.73-2.46) | <0.001 | 2.00 (1.68-2.39) | <0.001 | 1.72 (1.44-2.06) | <0.001 |
| **AD** |  |  |  |  |  |  |  |  |  |  |
| Control group | 159980 | 520 | 465308 | 1.12 (1.02-1.22) | ref |  | ref |  | ref |  |
| Osteoporosis group | 8147 | 61 | 20477.9 | 2.98 (2.28-3.83) | 1.81 (1.38-2.38) | <0.001 | 1.76 (1.34-2.32) | <0.001 | 1.50 (1.13-1.98) | 0.004 |
| **VD** |  |  |  |  |  |  |  |  |  |  |
| Control group | 159980 | 242 | 465613.4 | 0.52 (0.46-0.59) | ref |  | ref |  | ref |  |
| Osteoporosis group | 8147 | 38 | 20500.3 | 1.85 (1.31-2.54) | 2.80 (1.96-4.00) | <0.001 | 2.77 (1.94-3.96) | <0.001 | 2.07 (1.44-2.97) | <0.001 |
| **Further adjusting for antihypertensive, antidiabetic, lipid-lowering, and antiplatelet medications and proton pump inhibitors use** | | | | | | | | | | |
| **Dementia** |  |  |  |  |  |  |  |  |  |  |
| Control group | 166545 | 1185 | 481609 | 2.46 (2.32–2.60) | - |  | - |  | ref |  |
| Osteoporosis group | 9605 | 182 | 23813.9 | 7.64 (6.57–8.84) | - | - | - | - | 1.81 (1.53–2.15) | <0.001 |
| **AD** |  |  |  |  |  |  |  |  |  |  |
| Control group | 166545 | 543 | 482491.3 | 1.13 (1.03–1.22) | - |  | - |  | ref |  |
| Osteoporosis group | 9605 | 74 | 23964.7 | 3.09 (2.42–3.88) | - | - | - | - | 1.51 (1.16–1.97) | 0.002 |
| **VD** |  |  |  |  |  |  |  |  |  |  |
| Control group | 166545 | 252 | 482825.2 | 0.52 (0.46–0.59) | - |  | - |  | ref |  |
| Osteoporosis group | 9605 | 46 | 23992.5 | 1.92 (1.40–2.56) | - | - | - | - | 2.15 (1.52–3.04) | <0.001 |
| **Restricting the control group to older adults without osteoporosis based on BMD assessed by quantitative ultrasound** | | | | | | | | | | |
| **Dementia** |  |  |  |  |  |  |  |  |  |  |
| Control group | 19496 | 180 | 73561.6 | 2.45 (2.10–2.83) | ref |  | ref |  | ref |  |
| Osteoporosis group | 9605 | 182 | 23813.9 | 7.64 (6.57–8.84) | 2.63 (2.07–3.33) | <0.001 | 2.50 (1.97–3.17) | <0.001 | 1.86 (1.44–2.40) | <0.001 |
| **AD** |  |  |  |  |  |  |  |  |  |  |
| Control group | 19496 | 83 | 73706.3 | 1.13 (0.90–1.40) | ref |  | ref |  | ref |  |
| Osteoporosis group | 9605 | 74 | 23964.7 | 3.09 (2.42–3.88) | 2.27 (1.58–3.25) | <0.001 | 2.11 (1.47–3.02) | <0.001 | 1.54 (1.05–2.25) | 0.026 |
| **VD** |  |  |  |  |  |  |  |  |  |  |
| Control group | 19496 | 34 | 73760.7 | 0.46 (0.32–0.64) | ref |  | ref |  | ref |  |
| Osteoporosis group | 9605 | 46 | 23992.5 | 1.92 (1.40–2.56) | 4.26 (2.56–7.08) | <0.001 | 4.07 (2.44–6.79) | <0.001 | 2.70 (1.57–4.62) | <0.001 |

Abbreviations: AD, Alzheimer's disease; VD, vascular dementia; IR, incidence rate; CIs, confidence interval; HR, hazard ratio

Model 1: adjusted for age at baseline, sex, education, marital status and household registration;

Model 2: Model 1 plus smoking, regular exercise, body mass index category, and systolic blood pressure;

Model 3: Model 2 plus disease history, including hypertension, diabetes, dyslipidemia, stroke, cancer, chronic kidney disease, and depression.

### Table S11. Incident rates and Hazard ratios for all-cause dementia, AD, and VD for the osteoporosis patients and control groups when incident osteoporosis during follow-up was treated as a time-varying exposure

| **Outcome** | **N** | **No. of events** | **Person-years** | **IR (95% CI), per 1,000 person-years** | **Model 1** | | **Model 2** | | | **Model 3** | |
| --- | --- | --- | --- | --- | --- | --- | --- | --- | --- | --- | --- |
|  |  |  |  |  | HR (95% CI) | *P* | | HR (95% CI) | *P* | HR (95% CI) | *P* |
| **Dementia** |  |  |  |  |  |  | |  |  |  |  |
| Control | 166545 | 970 | 453414.5 | 2.14 (2.01-2.28) | ref |  | | ref |  | ref |  |
| Incident osteoporosis | 14843 | 215 | 28194.5 | 7.63 (6.64-8.72) | 1.66 (1.42-1.94) | <0.001 | | 1.65 (1.41-1.92) | <0.001 | 1.58 (1.35-1.85) | <0.001 |
| Prevalent osteoporosis | 9605 | 182 | 23813.9 | 7.64 (6.57-8.84) | 2.37 (2.01-2.81) | <0.001 | | 2.31 (1.96-2.74) | <0.001 | 1.96 (1.65-2.33) | <0.001 |
| **AD** |  |  |  |  |  |  | |  |  |  |  |
| Control | 166545 | 439 | 454058.2 | 0.97 (0.88-1.06) | ref |  | | ref |  | ref |  |
| Incident osteoporosis | 14911 | 104 | 284333.2 | 3.66 (2.99-4.43) | 1.57 (1.25-1.97) | <0.001 | | 1.56 (1.25-1.96) | <0.001 | 1.49 (1.19-1.88) | 0.001 |
| Prevalent osteoporosis | 9605 | 74 | 23964.7 | 3.09 (2.42-3.88) | 2.06 (1.59-2.67) | <0.001 | | 2.00 (1.54-2.59) | <0.001 | 1.68 (1.29-2.19) | <0.001 |
| **VD** |  |  |  |  |  |  | |  |  |  |  |
| Control | 166545 | 210 | 454289.9 | 0.46 (0.40-0.53) | ref |  | | ref |  | ref |  |
| Incident osteoporosis | 14943 | 42 | 28535.3 | 1.47 (1.06-1.99) | 1.60 (1.13-2.27) | 0.008 | | 1.60 (1.12-2.27) | 0.009 | 1.46 (1.03-2.09) | 0.035 |
| Prevalent osteoporosis | 9605 | 46 | 23992.5 | 1.92 (1.40-2.56) | 3.17 (2.25-4.45) | <0.001 | | 3.12 (2.22-4.38) | <0.001 | 2.28 (1.60-3.24) | <0.001 |

Abbreviations: AD, Alzheimer's disease; VD, vascular dementia; IR, incidence rate; CIs, confidence interval; HR, hazard ratio

Participants with incident osteoporosis contributed person-time to the control group until an osteoporosis diagnosis, after which they contributed to person-time in the incident osteoporosis group.

Model 1: adjusted for age at baseline, sex, education, marital status and household registration;

Model 2: Model 1 plus smoking, regular exercise, body mass index category, and systolic blood pressure;

Model 3: Model 2 plus disease history, including hypertension, diabetes, dyslipidemia, stroke, cancer, chronic kidney disease, and depression.

### Table S12. ICD-10 codes used in the present study

| **Diagnosis** | **ICD-10 codes** |
| --- | --- |
| Osteoporosis | M80-M81 |
| Dementia | F00-F03, F05.1, G30, G31.0, G31.8 |
| Alzheimer’s disease | G30, F00 |
| Vascular dementia | F01 |
| Osteoporotic fracture | M80, S12.0, S12.1, S12.2, S12.7, S22.0, S22.1, S32, S42.2, S42.3, S42.8, S42.9, S52.5, S52.6, S72.0-S72.2, T08 |
| Hypertension | I10-I15 |
| Diabetes | E10-E14 |
| Dyslipidemia | E78.0-E78.5 |
| Cancer | C00-C97 |
| Stroke | I61-I64, I69.1-I69.4 |
| Chronic kidney disease | N18-N19 |
| Depression | F32-F33 |

Abbreviations: ICD-10, International Classification of Diseases 10th Revision


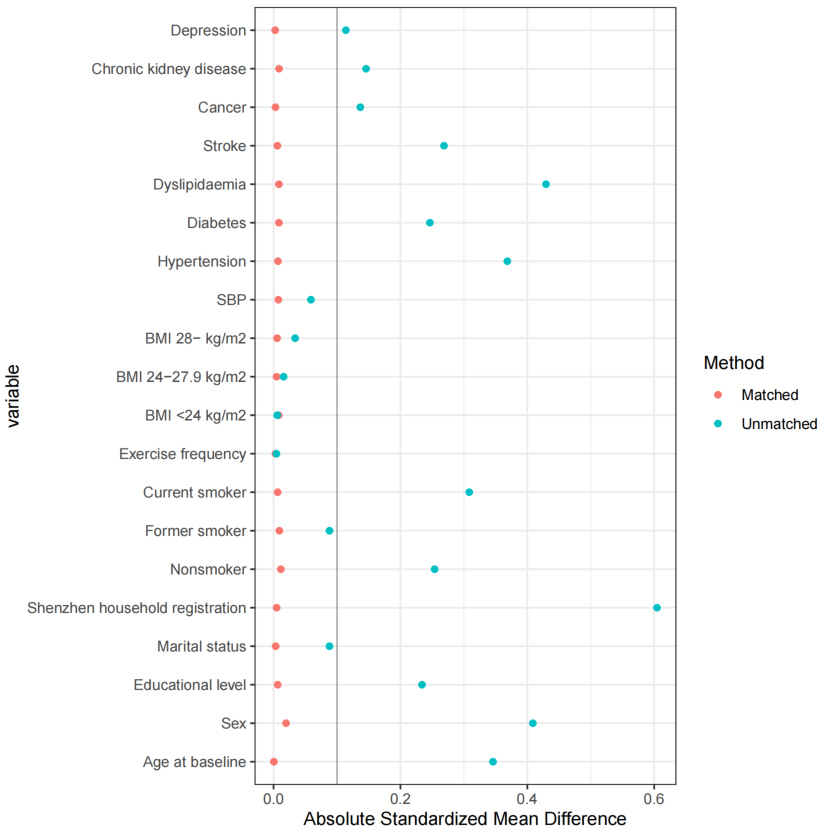


### Figure S1. Absolute standardized mean difference before and after propensity score matching

Abbreviations: BMI, Body Mass Index; SBP, Systolic Blood Pressure.

Propensity score matching was employed through a 1:5 match without replacement, using the nearest neighbor matching algorithm and a caliper of 0.1. The propensity scores were calculated by logistic regression, which accounted for age at baseline (±1 years), sex, education, marital status, household registration, smoking, regular exercise, BMI category, SBP, and disease history (including hypertension, diabetes, dyslipidemia, stroke, cancer, chronic kidney disease, and depression). A standardized mean difference of less than 0.1 was considered an indication of covariate balance.


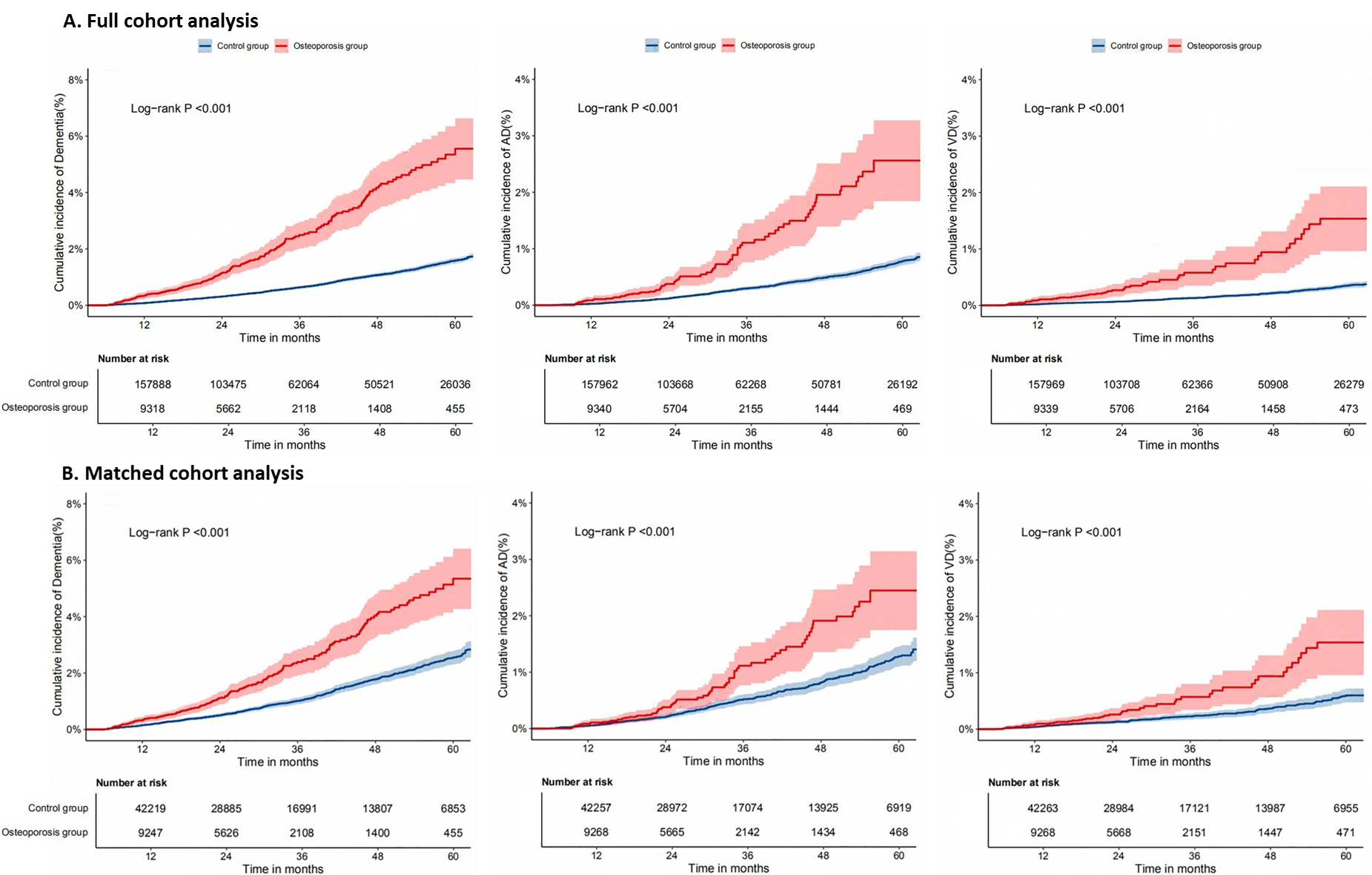


### Figure S2. Cumulative incidence of all-cause dementia, AD, and VD for the osteoporosis and control groups

Abbreviations: AD, Alzheimer's disease; VD, vascular dementia.


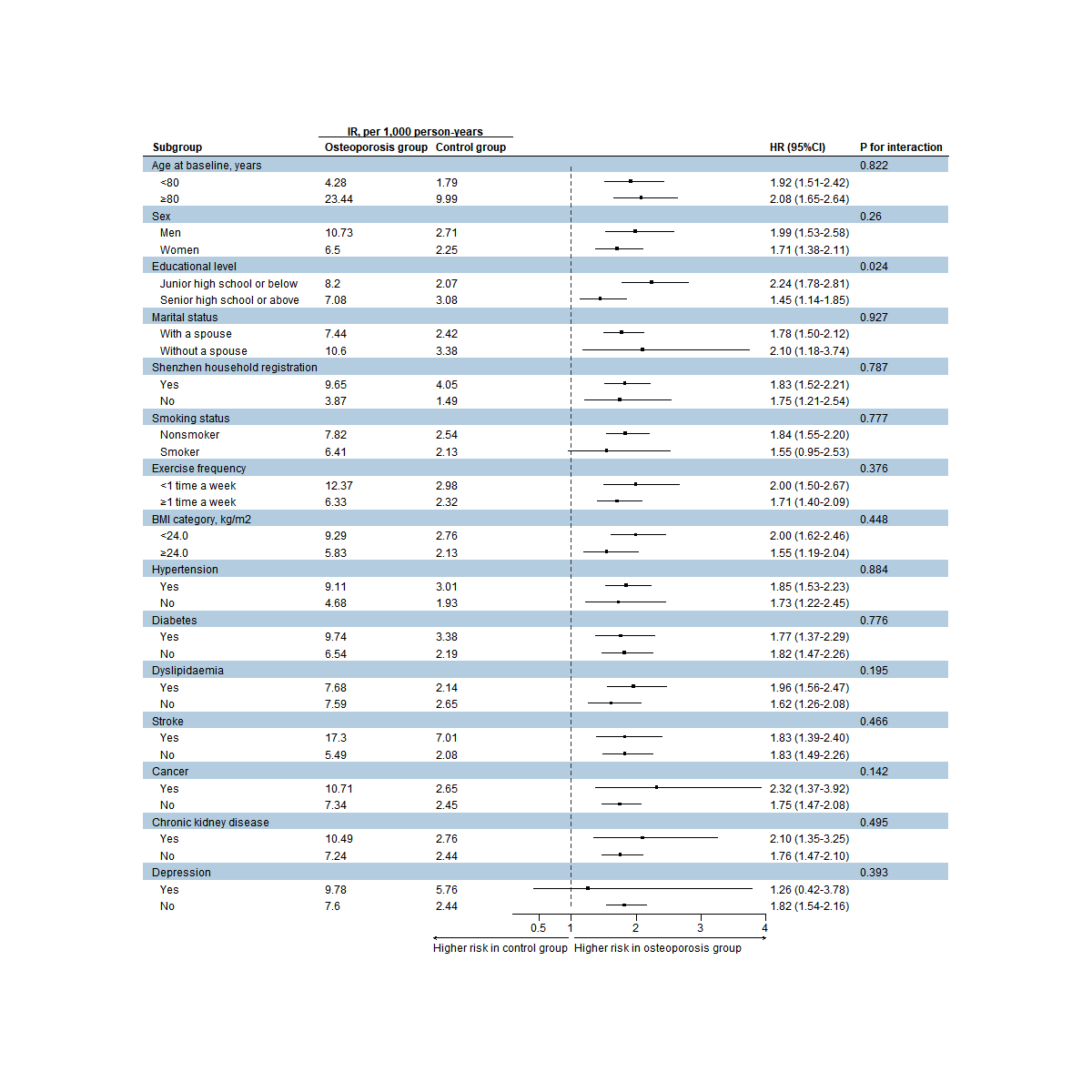


### Figure S3. Association between osteoporosis and all-cause dementia by subgroups

Abbreviations: BMI, body mass index; IR, incidence rate; CIs, confidence interval; HR, hazard ratio.

All models were adjusted for age at baseline, sex, education, marital status, household registration, smoking, regular exercise, body mass index category, systolic blood pressure, and disease history (including hypertension, diabetes, dyslipidemia, stroke, cancer, chronic kidney disease, and depression).


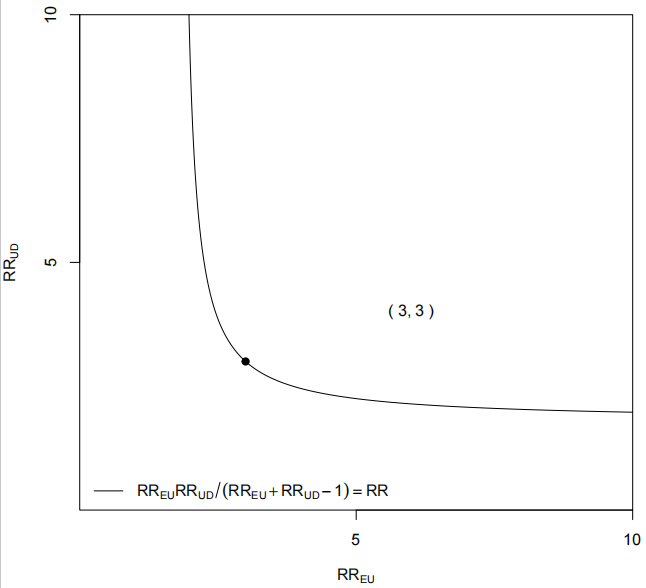


### Figure S4. The E-value analysis based on multivariable adjusted model for the observed association between osteoporosis and dementia

E-value was calculated to assess the impact an unmeasured confounder would need to have to explain the observed association between osteoporosis and dementia.

### Supplementary Methods

**Validity of Osteoporotic Fracture ascertainment**

To evaluate the validity of osteoporotic fracture ascertainment in the present study, we randomly sampled 200 osteoporotic fracture cases with linked medical records and imaging reports (including 100 inpatient and 100 outpatient cases). For each case, patient demographics, encounter date, physician notes, imaging reports (x-ray, CT or MRI), and surgical procedures were independently reviewed by two orthopedists. Any disagreement was resolved through consultation with senior orthopedists. The positive predictive value (PPV) with exact binomial 95% confidence intervals (CI) was calculated as the proportion of cases with osteoporotic fracture diagnoses that were confirmed through medical record review. Among the 200 cases, 3 inpatient cases and 8 outpatient cases were excluded due to insufficient clinical information for adjudication. The resulting PPVs were 95.8% (95% CI: 91.8%-98.2%) overall, 97.9% (95% CI: 92.7%-99.7%) for inpatient cases, and 93.5% (95% CI: 86.3%-97.6%) for outpatient cases.
